# Improved Stability and Brightness Following Iterative Redesign of a De Novo Biliprotein

**DOI:** 10.1021/acs.biochem.6c00166

**Published:** 2026-06-15

**Authors:** Felix S. Morey-Burrows, Tingxiang Yang, Woody Ahern, David Baker, Andrew Hitchcock, Graham J. Leggett, Jenny Clark, Aneika C. Leney, C. Neil Hunter

**Affiliations:** † School of Biosciences, 7315 University of Sheffield, Firth Court, Western Bank, Sheffield S10 2TN, U.K.; ‡ Institute for Protein Design, 7284 University of Washington, Seattle, Washington 98105, United States; § Department of Chemistry, University of Sheffield, Sheffield S3 7HF, U.K.; ∥ School of Mathematical and Physical Sciences, University of Sheffield, Hicks Building, Hounsfield Road, Sheffield S3 7RH, U.K.; ⊥ School of Biosciences, 1724 University of Birmingham, Birmingham B15 2TT, U.K.

## Abstract

Bilins, linear tetrapyrroles derived from heme catabolism, are ubiquitous across biological systems. The constraints imposed by binding to a protein confer valuable optical properties on bilins, which are used for sensing and harvesting light, and offer potential for fluorescence imaging, optogenetics, and biosensing. Recently, bilins have been used to evaluate methods for designing ligand binding sites, and novel biliproteins (BPs) that bind phycoerythrobilin (PEB) emerged from computational design using RoseTTAFold Diffusion All-Atom (RFDiffusionAA) and LigandMPNN. Here, we find much lower stability, extinction coefficients and fluorescence quantum yields for de novo designs compared to the native CpcA-PEB biliprotein, so we used a LigandMPNN-AlphaFold 3 (AF3)-Rosetta Relax pipeline to redesign one BP, C11, which can be implemented quickly and without in-house GPUs. The top three ligand confidence scores emerging from this procedure yielded C11-578, C11-620, and C11-756 redesigns that retain 50%, 52%, and 57% identities, respectively, with C11. AF3-Rosetta modeling of the BP redesigns predicted similar conformations of the PEB, decreased solvent-accessible surface area of the bilin binding site, an additional predicted hydrogen bond in C11-620, and a more rigid protein backbone. The new constraints on bilin binding improved the stability, absorption properties and fluorescence yields for C11-578, C11-620, and C11-756, and the brightness of the redesigns improved up to 13-fold, approaching the values for the native CpcA-PEB BP. Further design iterations can be used to create a range of absorbing and emitting BPs, including oligomeric assemblies housing internal energy cascades.

## Graphical abstract

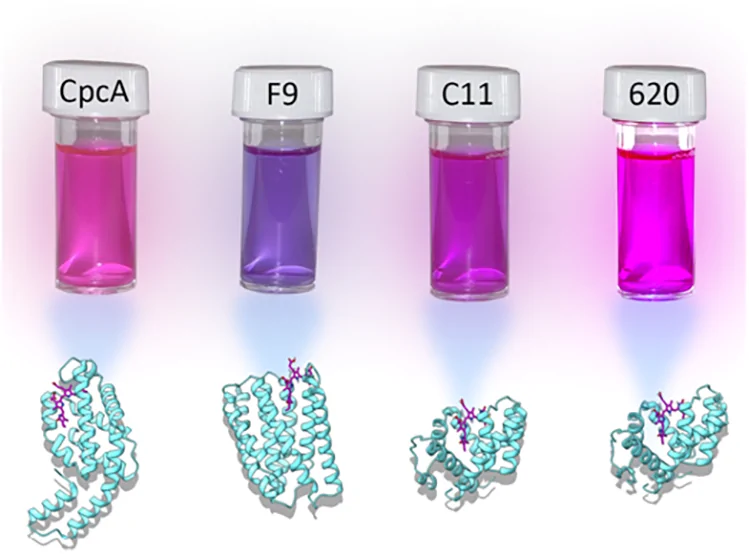

## Introduction

Bilins display limited optical properties in solution, but binding within protein scaffolds confers significantly enhanced absorption and fluorescence characteristics.^1^ The variety of bilins, and the strong optical effects exerted by binding to a wide range of proteins, offer a large number of functional options, exploited by diverse organisms for both light sensing^2,3^ and light harvesting.^2,4,5^ Thus, biliproteins (BPs) such as phytochromes are capable of responding to light and activating a downstream signal,^6^ while others are tuned for spectral range and high levels of absorption and fluorescence, which are ideal for harvesting light to drive charge separation in photosynthesis.^4^ For example, multiple bilin types bind to a series of proteins that oligomerize to form giant (0.47 to 18.7 MDa) membrane-extrinsic light-gathering arrays called phycobilisomes (PBSs). Cryogenic electron microscopy (cryo-EM) has been used to determine the structures of the major structural classes of PBS, reviewed in ref 4, showing how the strategic positioning of different phycobiliproteins creates an energy cascade that funnels excitations to charge-separating reaction centers.^7–9^ The remarkable advances in cryo-EM now show how PBSs can mediate photoprotection,^10^ and adapt to far-red light^11^ and changing light levels.^12^

Generally, light-harvesting bilins are covalently attached to bilin binding proteins via a thioether linkage to a cysteine residue.^13–16^ This post-translational modification is carried out by a bilin lyase, which recognizes a “CXRD” motif, orientating the bilin with respect to the cysteine to allow efficient and correct attachment.^13–16^ There are different bilin lyase proteins for different bilins, although there is some promiscuity between these lyase molecules, and indeed between bilin-binding proteins.^15^ Five major bilin pigments are utilized in sensing and harvesting light: phycoerythrobilin (PEB), phycocyanobilin (PCB), phytochromobilin (PΦB), phycourobilin (PUB) and phycoviolobilin (PVB).^2,17^ PEB, PCB, and PΦB are synthesized directly from biliverdin, which is made by linearizing the haem macrocycle using haem oxygenase. The other two pigments, PUB and PVB, are isomers of PEB and PCB, respectively, and are generated in tandem with their attachment to bilin binding proteins–i.e., the bilin lyase also has isomerase activity.^18^ PΦB is found in plant phytochromes, which mediate phototropism,^19^ while the remaining four bilins are involved in light harvesting.^8,17,20,21^ The PBSs of some organisms employ a mixture of these four bilins in order to optimize collection of solar energy for photosynthesis.^8^ Other organisms, for example the cyanobacterium *Synechocystis* spp. PCC 6803 use only PCB,^10^ likely dictated by the spectral quality in a given environment,^22,23^ while the red alga *Porphyridium purpureum* contains PUB, PEB, and PCB and is found in moist terrestrial environments, where red-light filtering by water is less common.^24^

Given the attractive optical properties of bilins and their ability to act as reporters of protein binding, the novel computational methodologies RoseTTAFold Diffusion All-Atom (RFDiffusionAA) and LigandMPNN^25,33^ were used to generate thousands of BP designs, based on the structure of the PCB chromophore in CpcA, a well-studied BP from *Synechocystis* sp. PCC 6803. Every design included the “CARD” motif required for recognition by the bilin lyase machinery.^25^ Our work employed the CpcE/F lyase, which natively attaches PCB to CpcA but has been found to display activity with a variety of bilins^25^ and has been used previously for the heterologous production of a range of BPs in *Escherichia coli*.^26,27^ A 96-well plate assay was devised to screen a subset of these designs, which employed an *E. coli* expression system also capable of synthesizing PEB and the CpcE/F lyase, based on earlier work.^26,27^ The screen identified promising proteins, denoted C11 and F9, that exhibited strong bilin binding *in vivo*, which validated the design methodology.^25^ Here, we use biochemical and spectroscopic methods to examine one of the designs, C11; fluorescence excitation measurements indicate a suboptimally defined PEB binding pocket, which could arise when this flexible tetrapyrrole pigment adopts a range of conformations that modify the coplanarity of the A, B, C, and D pyrrole rings, or which change electrostatic interactions or the extent of hydrogen bonding between the bilin and its binding site. C11 therefore provides a useful platform to iteratively redesign a ligand binding site, which in this case should improve the stability, extinction coefficient and fluorescence quantum yield. We developed a computationally efficient protein design pipeline–LigandMPNN-AlphaFold 3 (AF3)-Rosetta Relax–that produced three C11 redesigns with significantly enhanced stability and optical properties. Using AF3 and Rosetta Relax, we rationalized these improvements, attributing them to a tightened bilin-binding pocket. Our study offers key insights into improving small molecule binding through a computationally inexpensive method and provides a foundation for future synthetic biology studies of photosynthesis.

## Materials and Methods

### Protein Design Pipeline

The output from a previous design study^25^ was relaxed in Rosetta using the scoring function ref2015. A physical bond was introduced using ChimeraX to form the thioether linkage and a constraint file (cys_cyc_bond.cst) was used to apply a distance constraint of 1.8 Å with a standard deviation of 0.06 Å and a Gaussian distribution. A simple RosettaScripts (SolRelax.xml) file was used. This was relaxed 10 times, and the best scoring model was used for the next step.

LigandMPNN was run using a simple shell script (C11_new.sh), which allowed all the residues to change to any side chain (excluding cysteines), except for the “CARD” motif, generating 1000 variants.

The output of LigandMPNN is a FASTA file of sequences, each with an associated ligand confidence score (0–1). The sequence with the highest score was selected and used to run AF3 from the web server, which does not include PEB as a ligand and was therefore run as an apoprotein.

The output structure was aligned with the Rosetta relaxed C11 to manually dock the ligand in the binding site of the new variant. This was then relaxed in Rosetta as described for the original C11. This process was looped, passing the structure back through LigandMPNN, generating new variants, refolding with AF3 and relaxing with Rosetta until the ligand confidence scores showed no further improvements. The best scoring variants from the previous step were tested in the wet lab. A schematic flowchart outlining this pipeline can be found in Figure S14.

### Buffers

Size exclusion chromatography (SEC) and all analyses were performed in 500 mM NaCl, 50 mM HEPES at pH 7.6, unless otherwise stated. Immobilized Metal Affinity Chromatography (IMAC) was carried out on benchtop columns with 2 mL resin and with buffers the same as for SEC with imidazole added as follows: binding buffer: 10 mM imidazole; wash buffer: 50 mM imidazole; elution buffer: 450 mM imidazole.

### Construction of Plasmids

Synthetic genes for the redesigns with N-terminal His_8_ tags followed by tobacco etch virus (TEV) protease cleavage sites, a C-terminal stop codon and N-terminal/C-terminal NdeI/XhoI restriction sites were purchased as gBlocks from Integrated DNA Technologies. DNA was digested with NdeI/XhoI restriction enzymes and ligated into the expression plasmid pET21a. The ligations were transformed into chemically competent *E. coli* JM109 (New England Biolabs) and plated on LB agar supplemented with 100 μg/mL ampicillin. For each design, single colonies were grown overnight in 5 mL LB medium supplemented with 100 μg/mL ampicillin. Plasmids were extracted using the FastGene Plasmid Mini Kit (Nippon Genetics) and sequence of inserts verified (Eurofins Scientific). pET21a plasmids with an N-terminal His_6_ tag followed by a TEV protease cleavage site were used for the expression of the original C11, F9 designs, and CpcA.^25^

### Protein Expression and Purification

For each design, the sequence-verified plasmids were transformed into chemically competent *E. coli* BL21­(DE3) harboring a pCOLADuet-*cpcEF-pebS*-*hox1* expression plasmid^31^ capable of synthesizing PEB, and plated onto LB agar with 100 μg/mL ampicillin. A single *E. coli* colony was grown in 5 mL LB with 100 μg mL^–1^ ampicillin overnight at 37 °C. Overnight cultures were used to inoculate 1 L cultures of Terrific Broth (TB), and grown at 37 °C with shaking at 180 rpm for 16–18 h. Bacteria were harvested and resuspended in 500 mM NaCl, 10 mM imidazole, 50 mM HEPES buffer at pH 7.6, ∼0.01 mg/mL DNase (Sigma-Aldrich), and ∼0.1 mg/mL lysozyme (Sigma-Aldrich). Bacteria were lysed by sonication and centrifuged at 21,000*g* for 30 min. Soluble fractions were purified using IMAC gravity columns packed with Ni-NTA agarose resin (Qiagen) at room temperature. Columns were washed with a buffer containing 50 mM imidazole and proteins were eluted with 450 mM imidazole buffer. Proteins were further purified by SEC using an ÄKTA Go FPLC instrument with a Superdex 75 Increase 10/300 GL column (GE Healthcare Life Sciences), in 500 mM NaCl, 50 mM HEPES at pH 7.6.

### Ultraviolet Visible (UV–Vis) Absorption Spectroscopy

UV–visible absorption spectroscopy was carried out using a Cary 60 spectrophotometer (Agilent) in UV-permissive polystyrene or quartz cuvettes with a 1 cm path length. All spectra corrected with an appropriate blank and diluted below an absorbance of 0.5.

### Native Mass Spectrometry

Prior to native mass spectrometry analysis, all BP samples were buffer exchanged into 100 mM ammonium acetate pH 6.8 using Amicon Ultra 0.5 mL concentrators with a 3 kDa molecular weight cutoff filter (Merck Millipore). All BPs were analyzed at a concentration of 5 μM. Native mass spectrometry experiments were performed on an Orbitrap Ascend Structural Biology mass spectrometer (Thermo Fisher Scientific) using a nanoelectrospray ionization source fitted with gold coated borosilicate glass capillaries pulled in-house (P-1000, Sutter Instrument). The instrument was calibrated with FlexMix. Positive ionization mode was used with a capillary voltage set between 1.2 and 1.4 kV. Mass spectra were acquired in intact protein mode at high pressure, with the in-source fragmentation set to zero. Ions were detected in the Orbitrap with a mass range set to 500–6000 *m*/*z*, resolution set to 15,000, Automatic Gain Control 100%, and the micro scan count set to 10. All spectra were acquired for >1 min and the average spectra over this time scale reported.

Native spectrometry data was processed using XCalibur v4.2 (Thermo Fisher Scientific) and deconvoluted using Unidec. Theoretical masses of the unmodified BPs were calculated from the amino acid sequences. The modified mass was adjusted to include the addition of 1 PEB chromophore (+586.7 Da). The CpcA and C11 protein masses included the loss of the initiator methionine (−131.2 Da), a modification commonly observed during bacterial protein expression. The percentage errors between the theoretical and experimentally determined molecular weights were used to verify the presence of the unmodified and modified BPs (Table S1). Note, the most abundant species corresponded to proteins with a ∼60 Da mass adduct. To calculate the percentage modification for each protein, the relative abundance of all bilin-modified *m*/*z* peaks corresponding to the intact protein were summed and expressed as a percentage of the sum of the unmodified and modified protein *m*/*z* peaks.

### Fluorescence Spectroscopy (Emission and Relative Yield)

Samples were diluted such that the absorbance of the sample was between 0.09 and 0.1, measured using a Cary 60. 1 cm by 1 cm cuvettes were used for absorption and fluorescence measurements. Samples were excited from 520 to 530 nm, and emission spectra were recorded from 535 to 700 nm. Measurements were made on a Fluorolog2 fluorimeter (Horiba) with excitation from a xenon lamp (Osram).

CpcA-PEB was used as the reference fluorescent protein as its absolute quantum yield of fluorescence yield is known. An absorptance spectrum was calculated, to convert logarithmic absorbance into linear absorptance (eq 1).

1 $$\mathrm{Absorptance}=1-10^{-\mathrm{Absorption}}$$

The area under the absorptance graph that was excited during fluorescence experiments was calculated and compared to the area under the emission spectrum (eq 2). Setting the output from CpcA-PEB to 100%, the relative yields of proteins were calculated (eq 3).

2 $$Fluorescence\;per\;absorp\,\tan ce=\frac{\int_{\lambda_{Max}}^{800nm}Fluorescence\;spectrum}{\int_{\lambda_{Max}-5nm}^{\lambda_{Max}}Absorp\,\tan ce\;spectrum}$$

3 $$\mathrm{Relative}\;\mathrm{fluorescence}\;\mathrm{yield}\;\%=\frac{{\mathrm{Fluorescence}\;\mathrm{per}\;\mathrm{absorptance}}_{\mathrm{Sample}}}{{\mathrm{Fluorescence}\;\mathrm{per}\;\mathrm{absorptance}}_{\mathrm{CpcA}‐\mathrm{PEB}}}$$

Integrals were approximated using Simpson’s rule (eq 4).

4 $$\mathrm{Area}=\int_{b}^{a}f\left(x\right)dx\approx \frac{\Delta x}{3}\left(y_{0}+4y_{1}+2y_{2}+4y_{3}+2y_{4}+...+4y_{n-1}+y_{n}\right)$$

where $\Delta x=\frac{b-a}{n}$.

### Fluorescence Spectroscopy (Excitation)

Samples were diluted such that the absorbance of the sample was between 0.09 and 0.1, measured using a Cary 60. 1 cm by 1 cm cuvettes were used for absorption and fluorescence measurements. Excitation was carried out in 1 nm intervals. The range changed per sample but was generally measured from 400 nm to the bilin absorption maximum. Fluorescence was monitored continuously at the fluorescence emission maximum (+5 nm). Assuming absorption from the maximum contributes 100% to fluorescence emission, the excitation spectra were normalized to equal 1 at the maximum absorptance peak. These normalized excitation spectra were overlaid with the calculated absorptance spectrum to provide information on relative fluorescence contribution of each wavelength of absorption. Measurements were made on a Fluorolog2 (Horiba) with a xenon lamp (Osram).

### Transient Absorption (TA) Spectroscopy

Transient absorption spectra were recorded using a commercial spectrometer (Helios, Ultrafast Systems) outfitted with a Ti:sapphire seed laser (MaiTai, Spectra-Physics). The output from the seed laser was amplified in a Ti:sapphire regenerative amplifier (Spitfire Ace PA-40, Spectra-Physics) to deliver 800 nm pulses (10 kHz, 1.2 mJ, 40 fs nominal FWHM). Tunable narrowband pump pulses at 565 and 572 nm were generated in an optical parametric amplifier (TOPAS Prime, Light conversion). The pump was modulated by an optical chopper, lowering the repetition rate to 5 kHz, and its average energy was adjusted to 22.74 J/m^2^ at the sample position with a variable neutral density filter. Supercontinuum white-light probe pulses were generated by focusing part of the 800 nm beam through a continuously translating CaF_2_ crystal. The pump and probe polarizations were set to the magic angle (54.7°). After transmission through the sample, the probe beam was spectrally dispersed with a grating and detected with a CMOS sensor. TA data were processed using background subtraction and wavelength-dependent chirp correction. The sample was prepared in a buffer solution with an OD of 0.4 at the excitation wavelengths.

### Fluorescence Lifetime Measurements

The fluorescence lifetime kinetics of samples were measured on a home-built time-resolved fluorescence setup equipped with a single-photon hybrid photodetector (Becker & Hickl, HPM-100–50). A 485 nm picosecond diode laser (PicoQuant, LDH-D-C-485) with a repetition rate of 50 MHz was applied for lifetime measurements. Fluorescence emission detection was filtered through a 550 nm long-pass filter. The laser beam was focused on the sample surface to a diffraction-limited spot using a 20×/0.5 HD air objective (ZEISS, EC Epiplan-NEOFLUAR). The modulation of the laser was synchronized with a time-correlated single-photon counting module (Becker & Hickl, SPC-150). The instrument response function (IRF) of the system is ∼160 ps. Data were processed with Origin 2024.

### PEB Purification

A gene encoding C11–620 C111A was coexpressed with the bilin biosynthesis machinery, as with the other designs. This variant was subsequently purified via IMAC yielding a red-shifted BP due to absence of the thioether linker. The protein was buffer exchanged into water using a PD-10 column, concentrated and then diluted in ∼10 volumes of MeOH and the aggregated protein pelleted at 4000*g*, leaving a pink supernatant. This was dried in a rotary evaporator and resuspended in minimal MeOH to ∼0.5 mM PEB, as determined using an extinction coefficient at 591 nm in MeOH/5% HCl of 25.2 mM^–1^ cm^–1^.^28^

### Reconstitution/Binding Affinity Experiments

SEC-purified proteins were treated with 5 mM DTT and buffer exchanged to remove DTT. Protein solutions were diluted to 1 μM and added to a fluorescence imaging plate (Greiner, PS 96-well, F-bottom) in triplicate along with a buffer only control. A range of concentrations from 30 nM to 15 μM were added to the protein or buffer solutions with a maximum MeOH concentration of 2.5%. The plate was left for 1 h to allow binding and thioether bond formation, after which fluorescence was measured using a Hidex Sense plate reader with excitation at 525 nm (+/10 nm) and measured at 575 nm (+/–10 nm). Emission signal from ligand in buffer was subtracted from ligand + buffer fluorescence, which was plotted against PEB concentration and fit with a one-site specific binding formula: (*B*_max_ × *x*)/(*K*_*d*_ + *x*).

### Rosetta Soluble Protein Minimization

PDBs of relevant protein–ligand complexes were parsed through Rosetta Parser Protocol, with relevant ligand parameter files (.params) and a RosettaScript (.xml) for relaxation with 10 decoys constructed each time, and the best output based on scoring using ref2015 was selected for further use in protein design or analysis. See ref 29 for more information. Scores are given in Rosetta Energy Units (REU).

## Results

### Evaluation of the Original BP Designs in Relation to the Native Phycobilisome Component, CpcA

Genes encoding the His_6_-tagged protein designs C11 and F9 ^25^ and the native BP CpcA (the protein sequences are shown in Figure S1) were heterologously expressed in *E. coli* BL21(DE3), previously transformed with pCOLA bearing genes encoding heme oxygenase (HOI), PEB synthase (PebS) and the bilin lyase system, CpcEF. Thus, this background produces the PEB bilin, which is covalently attached to C11, F9 or CpcA. The respective BPs were purified using IMAC, with useful fractions easily identified by their vivid colors. Absorption spectra showed a 50 nm spectral range for PEB bound to the C11, F9, and CpcA proteins (Figures S2 and 1). A single major elution peak was observed in SEC, suggesting that these designs are in a single oligomeric state (Figure S3) and, given the assumption that CpcA-PEB is a monomer, also likely monomeric.

**Figure 1:**
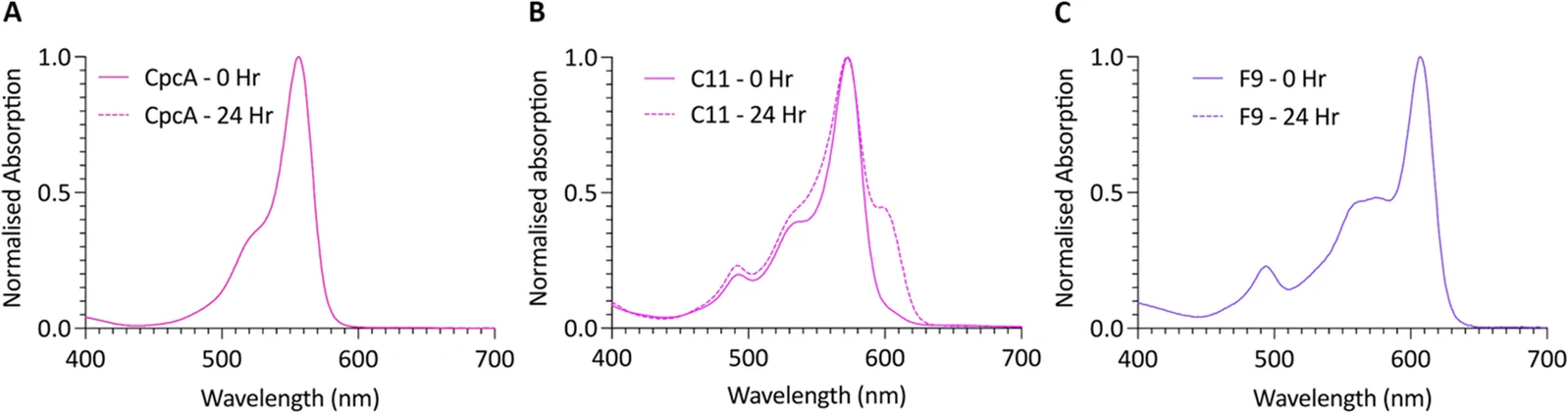
Absorption spectra illustrating the stability of native and designed biliproteins following storage at 4 °C for 24 h. (A) CpcA control, (B) C11 and (C) F9. All samples were diluted to an absorbance maximum between 0.1 and 0.5, corresponding to protein concentrations between 20 and 60 μM. Measurements were carried out in 500 mM NaCl, 50 mM HEPES at pH 7.6.

Attempts to unfold the protein/change the bilin environment for the C11 and F9 designs using either 8 M urea, 6 M guanidinium hydrochloride, or heating to 95 °C were unsuccessful (data not shown). However, some instability was observed upon storage at 4 °C, but only for C11 (Figure 1B), where an absorption feature appears at around 600 nm, and another intensifies at ∼540 nm. The heat stability of the bilins within these BPs prevented the separation and quantification of the bilin and protein components so accurate determination of the BP extinction coefficient proved problematic, and a computational approach was adopted based on,^30^ reported to have an error margin of ±5%. Using this approximate value for the protein extinction coefficient, and the assumption of one bilin per apoprotein, a corresponding bilin extinction coefficient was calculated for each design. The calculated extinction coefficients at the bilin absorption maxima for CpcA, C11, and F9 were 89,101, 8,438, and 6,806 M^–1^ cm^–1^, respectively. Given that protein binding sites are known to confine bilins and confer enhanced absorption and fluorescence characteristics these large differences in extinction coefficients suggest that the BP designs are far less effective than the native CpcA in constraining the bilin. An alternative explanation is that the bilin binding site occupancy in these designs was lower than that in CpcA, assuming 100% occupancy in CpcA, which would imply that approximately one-third of the binding sites were occupied in C11. However, the actual situation is likely more complex, involving a combination of both reduced occupancy and decreased bilin constraints. We used mass spectrometry to quantify bilin binding site occupancy (see later Results section).

These limitations in terms of absorption were also reflected in the fluorescence properties of the designed BPs. By comparison with the absolute fluorescence yield (0.67) of CpcA-PEB,^31^ the absolute fluorescence yields for C11-PEB and F9-PEB were determined to be 0.31 and 0.11, respectively. Brightness $\left(\frac{Q_{Y}\times \varepsilon }{1000}\right)$ was subsequently calculated, yielding values of 59.7, 2.6, and 0.75 for CpcA-PEB, C11, and F9, respectively, in comparison to a brightness of 19.8 for GFP, for example.^32^

Unlike CpcA, the C11 and F9 designs show a discrepancy between their absorptance and fluorescence excitation spectra (Figure 2). This suggests that a portion of the absorbed energy is not emitted as fluorescence. Such a mismatch may indicate the presence of multiple protein populations harboring distinct bilin conformations.

**Figure 2:**
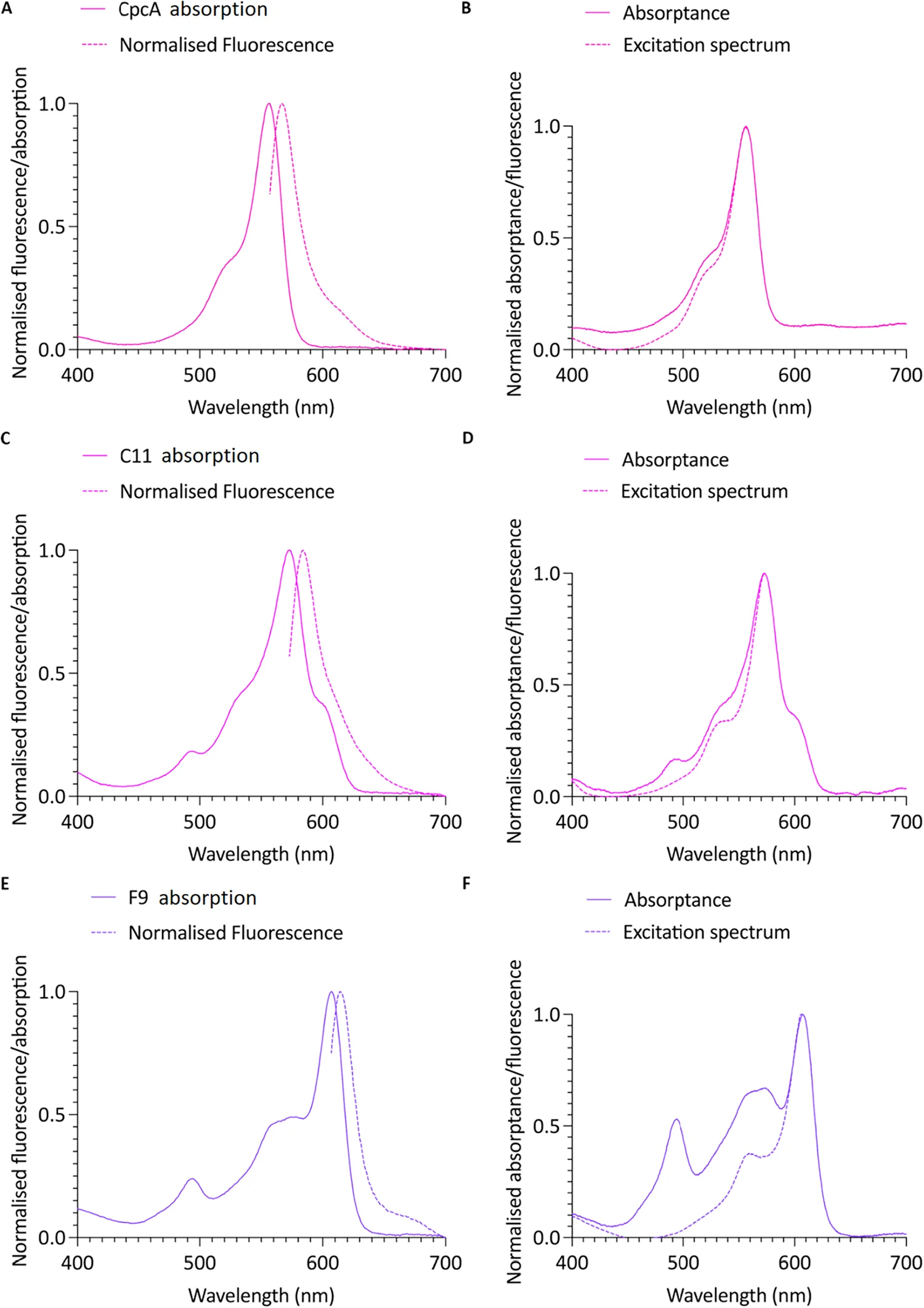
Absorption and fluorescence spectroscopy of native and designed biliproteins. (A, B) CpcA, (C, D) C11, and (E, F) F9. The left panels show normalized absorbance/fluorescence emission spectra, and the right panels show absorptance/excitation spectra.

In summary, the C11 and F9 designs exhibit neither the desired stability nor suitable spectroscopic properties for downstream applications so a redesign pipeline was used to investigate the possibility of improvements, focusing on C11.

### Using a LigandMPNN-AF3-Rosetta Redesign Pipeline to Improve C11

A novel pipeline was implemented, based on a combination of LigandMPNN,^33^ the AF3 server^34^ and Rosetta Relax;^35^ a flowchart outlining the process is highlighted in Figure S14. The two confidence outputs from LigandMPNN, namely overall confidence and ligand confidence, are expressed as numbers ranging from 0 to 1 and reflect the probability LigandMPNN assigns to a given sequence folding into the desired structure with a ligand bound in the specified conformation. Since the aim was to improve the bilin binding site, ligand confidence was chosen as the optimized parameter. First, a Rosetta relaxed model of C11 was generated with the bilin covalently bound to the thioether bilin linker within the predicted binding site. This structure was then parsed into LigandMPNN and only the “CXRD” motif was fixed (“CARD” in this case), while the amino acid identities of the remaining residues of the C11 sequence were allowed to change, generating 1000 sequences.

The sequence with the highest ligand confidence was folded with AF3 but, due to the lack of PEB (or other bilins) in the AF3 server, PEB was manually redocked into the structure in ChimeraX,^36^ the covalent thioether linker reintroduced, and the structure was once again relaxed in Rosetta; this process was iterated until the ligand confidence showed no further improvement. The final iteration lowered the ligand confidence score (Figure S4), so the top three ligand confidence sequences from iteration 6, designated C11-578, C11-620 and C11-756, were chosen for biochemical analysis; the respective ligand confidences were 0.6788, 0.6679, and 0.6672. The aligned sequences (Figure 3) show that C11-578, C11-620 and C11-756 retain 50.0%, 52.0% and 56.7% identity relative to the original C11 design. Interestingly, the region with least identity is the helix (residues ∼60–70) facing the bilin molecule on the other side of the thioether linker, suggesting that C11 presented some major incompatibilities with PEB in this location (see Figures S11 and S12), which compromised bilin site occupancy, extinction coefficients and fluorescence yields (see previous section). Additionally, residues 26–41 appear to have low pLDDT scores which have been fixed in C11–620 (Figures S11 and S12). Given that all but four of the C11 residues were allowed to vary, the outputs were all quite similar, with 83% identity between C11–578 and C11–620 and 87% identity between C11–756 and C11–578, likely imposed by the requirement for high confidence in bilin binding.

**Figure 3:**
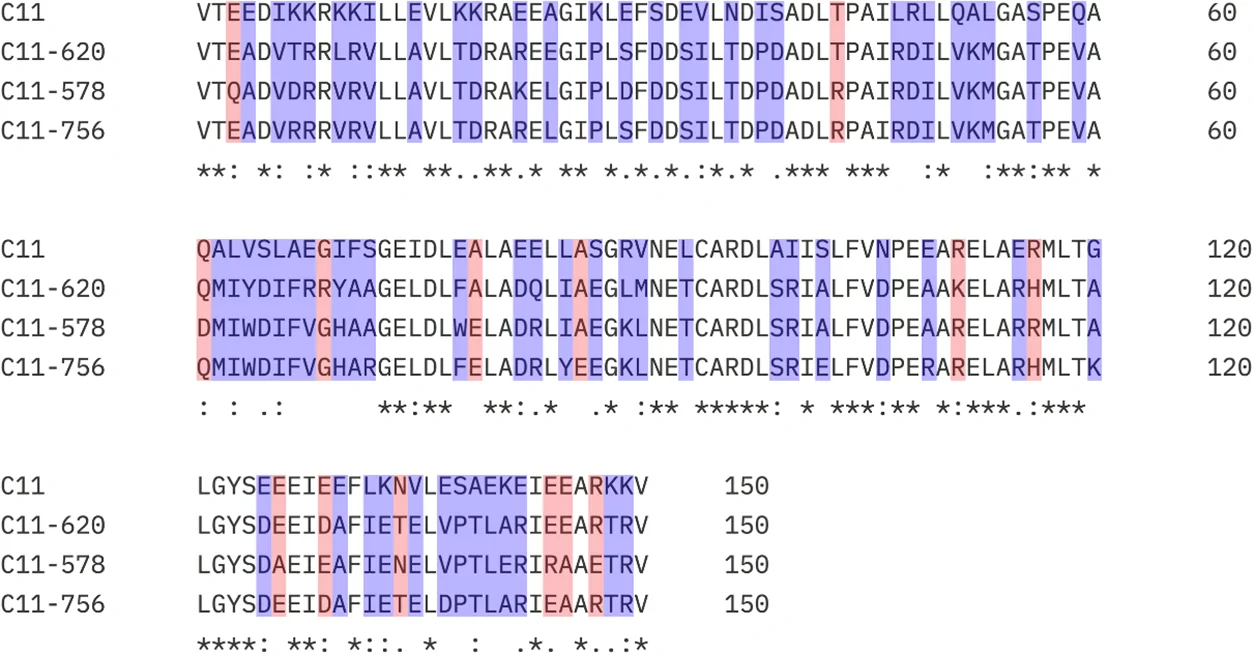
Protein sequences of the redesigns compared to C11. “*” = fully conserved residues; “:” = highly conserved residues; “.” = semiconserved residues; “ “ = non-conserved mutation. Colored highlights represent positions changed from the initial sequence; red indicates positions changed in some redesign sequences, while blue indicates the position is mutated across the 620, 578, and 756 redesigns.

### Characterization of Native, C11 and Redesigned BPs Using Mass Spectrometry

Synthetic gene fragments encoding redesigned BPs with a TEV-cleavable N-terminal His_8_ tag were inserted into the pET21a expression vector. Plasmids from the initial screen were used for preparation of CpcA and C11.^25^ Following preliminary trials, strains containing plasmids for producing designed BPs were grown overnight in 500 mL of Terrific Broth (TB) medium at 37 °C without induction, alongside C11 and CpcA controls. After pelleting the cells, lysis and clarification, the recombinant proteins in the supernatants were purified via IMAC. The native BP CpcA was also prepared in this manner, and further purified by SEC. There were only minor differences in the elution volume (Figure S5), showing that the new designs (all ∼19 kDa) appear to be monomeric; absorption spectra for the purified redesigns were similar, with only minor (2–3 nm) spectral shifts (Figure S6), and the spurious 490 nm peak in C11 is also almost absent from the redesigns. The full width at half-maximum (FWHM) values for C11-578, C11-620 and C11-756 (all 27 nm in Figure S6) show that the new designs display a more homogeneous bilin absorption relative to the original C11 (32 nm); for comparison the FWHM for CpcA is 28 nm (Figure S2 and Table 1). The Stokes shift for all the C11 variants and CpcA is 11 nm, and for F9 it is 7 nm.

**Table 1:** Absorption and Fluorescence Parameters for the Native CpcA, the C11 Design and the C11-578, C11-620, and C11-756 Redesigns^a^

BP	Bilin extinction coefficient at λ_max_ (M^–1^ cm^–1^)	Absolute FL yield	Relative FL yield (% CpcA)	Solvent-accessible surface area (Å^2^)	Brightness	Absorption FWHM (nm)	FL emission FWHM (nm)
CpcA	122,056	0.67	100.0	914	79.6	28.0	27.5
C11	9,699	0.31	46.1	941	3.5	32.0	29.0
578	14,317*	0.62	93.0	930	9.1	27.0	26.0
620	49,279	0.92	137.2	932	46.5	27.0	25.0
756	45,679*	0.68	101.8	936	31.9	26.5	25.0

a The relative fluorescence (FL) yields are scaled according to the value of 0.67 for CpcA-PEB reported in ref 31. * Mass spectrometry was not carried out on C11-578 or C11-756, so these extinction coefficients are based on the assumption of similar chromophorylation to C11-620.

We encountered problems in estimating occupancy of the bilin binding sites in native and C11 proteins, and assumptions of complete occupancy seemed unrealistic, affecting calculations of extinction coefficients for bound bilins. To overcome these issues, we used native mass spectrometry to quantify the levels of chromophorylation for native and designed BPs. Native mass spectrometry is a nondenaturing method that is designed to preserve protein structure and its noncovalent interactions from solution through to detection.^37^ The native mass spectra of the CpcA, C11 and C11-620 BPs are shown in Figure 4a–c. In all cases, two abundant charge state distributions are observed, one corresponding to the monomeric apoprotein, and the other to the corresponding protein with one bilin attached. Notably, the percentage of BP holoprotein in relation to the total was fairly consistent and ranged from ∼73–87%. Despite having the lowest extinction coefficient, C11 shows the highest bilin occupancy at 87%, while C11-620 and CpcA are 77% and 73%, respectively (Figure S15). Thus, we do not ascribe the different absorption and fluorescence properties of the native, designed and redesigned BPs to variable occupancy of the bilin binding sites. The fact that none of these numbers approached 100% reflects shortcomings in bilin production and/or ligation of PEB to the CARD motif in our *E. coli* expression system.

**Figure 4:**
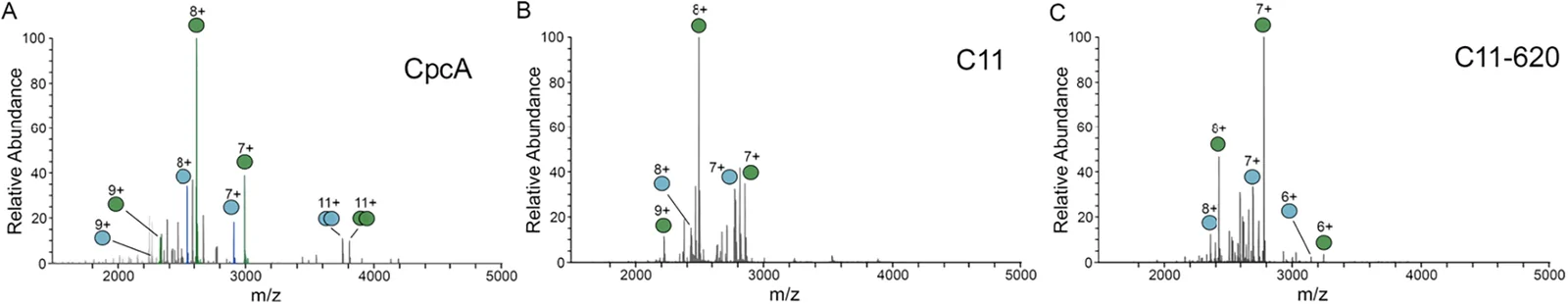
Native mass spectrometry of CpcA, C11, and the C11-620 redesign. (A) CpcA, (B) C11 and (C) C11-620. Peaks corresponding to different charge states of CpcA, C11, and C11-620 are colored in blue and green for the unmodified and singly-PEB bound proteins, respectively. Gray peaks correspond to protein contaminants. Note that the main peaks observed correspond to the theoretical protein sequence with a 58–62 Da mass adduct (Table S1). See Figure S15 for deconvoluted mass spectra.

The extent of chromophorylation allows calculations of extinction coefficients for protein-bound bilins, which are 122,056, 9,699 and 49,279 M^–1^ cm^–1^ for CpcA, C11 and C11–620, respectively (Table 1). Given the very similar % bilin occupancies these widely diverging extinction coefficients reflect large differences between the bilin binding environments for these proteins. The data in Table 1 show that the C11-578, C11-620 and C11-756 redesigns surpass the original C11, with C11-620 the best. Consistent with the absorption FWHM values, the new designs displayed narrower fluorescence emission bands (Figure 5, Figure 6) with respect to both the original C11 and the native CpcA (Table 1), supporting the idea that the redesigned binding sites impose more constraints on the conformations of PEB. Calculations of the fluorescence yields relative to CpcA, for which the absolute yield is known (0.67;^31^) showed marked improvements of all the redesigns with respect to C11. The native BP CpcA-PEB, with a brightness of 79.6, outperforms the de novo proteins (Table 1) but the values for C11–620 (46.5) and C11–756 (31.9) represent marked improvements relative to the initial design, C11, which has a low brightness of 3.5. The solvent-accessible surface area (SASA–Table 1) of the bilin in the Rosetta relaxed structure of each design was calculated (C11-578:930 Å^2^; C11-620:932 Å^2^; C11-756:936 Å^2^) and all displayed improvements when compared to the starting design, C11 (941 Å^2^), broadly consistent with a tightened binding site improving extinction coefficients and fluorescence yields. Fluorescence excitation spectra of C11 and the redesigns were recorded (Figure S8), measuring emission at 630 nm and exciting from 400 to 620 nm, in 1 nm increments. Unlike C11, the redesigns exhibit close correlation of the excitation (blue dashed lines) and absorptance spectra (red lines), so all the PEB absorption contributes to the fluorescence emission. The stability of C11 and redesigned proteins was analyzed following 24 h storage at 4 °C as for Figure 1. In contrast to C11, very few changes were observed for the new designs (Figure S9), demonstrating increases in stability consistent with improved brightness.

### Measurements of Binding Affinities for PEB in the Designed C11 and Redesigned C11-620 BPs

Purified PEB bilin in MeOH was reconstituted into bilin-free apoproteins in buffer, in order to measure the dissociation constants for this ligand for the respective binding pockets. This was not possible for the native CpcA, however, suggesting that the bilin must be loaded into this native protein during the folding process *in vivo*. Consistent with this, mutation of the cysteine residue in CpcA involved in covalent attachment of the bilin abolishes binding of PEB, but the analogous cysteine to alanine mutant of C11-620 still allows PEB to bind. Thus, PEB does not appear to bind during folding of the protein designs. In its bound state the PEB reconstituted into C11 and C11-620 acquires enhanced absorption, which is red-shifted because the usual process of covalently binding to a cysteine blue-shifts the absorption. Thus, in the absence of the cysteine the 520 nm shoulder shifts to ∼550 nm and the 570 nm peak moves to ∼600 nm (Figure S7).

Titrating PEB into solutions of C11 and C11-620 proteins yielded a series of increasingly fluorescent reconstitutions, as the protein binding site was gradually loaded with the initially poorly emitting bilin. Figure 5 shows the concentration dependence of bilin fluorescence emission, and the data for C11-620 allow calculation of the dissociation constant (*K*_D_), which is 1.41 ± 0.15 μM. However, the data for C11 suggest a weaker binding affinity beyond the tested concentrations; the poor solubility of PEB in aqueous solutions limited its use at higher concentrations, so the binding affinity of C11 could not be determined. Nevertheless, these data show that the C11-620 redesign has a much-improved binding affinity for PEB with respect to C11, highlighting the utility of the protein redesign pipeline outlined in this work.

**Figure 5:**
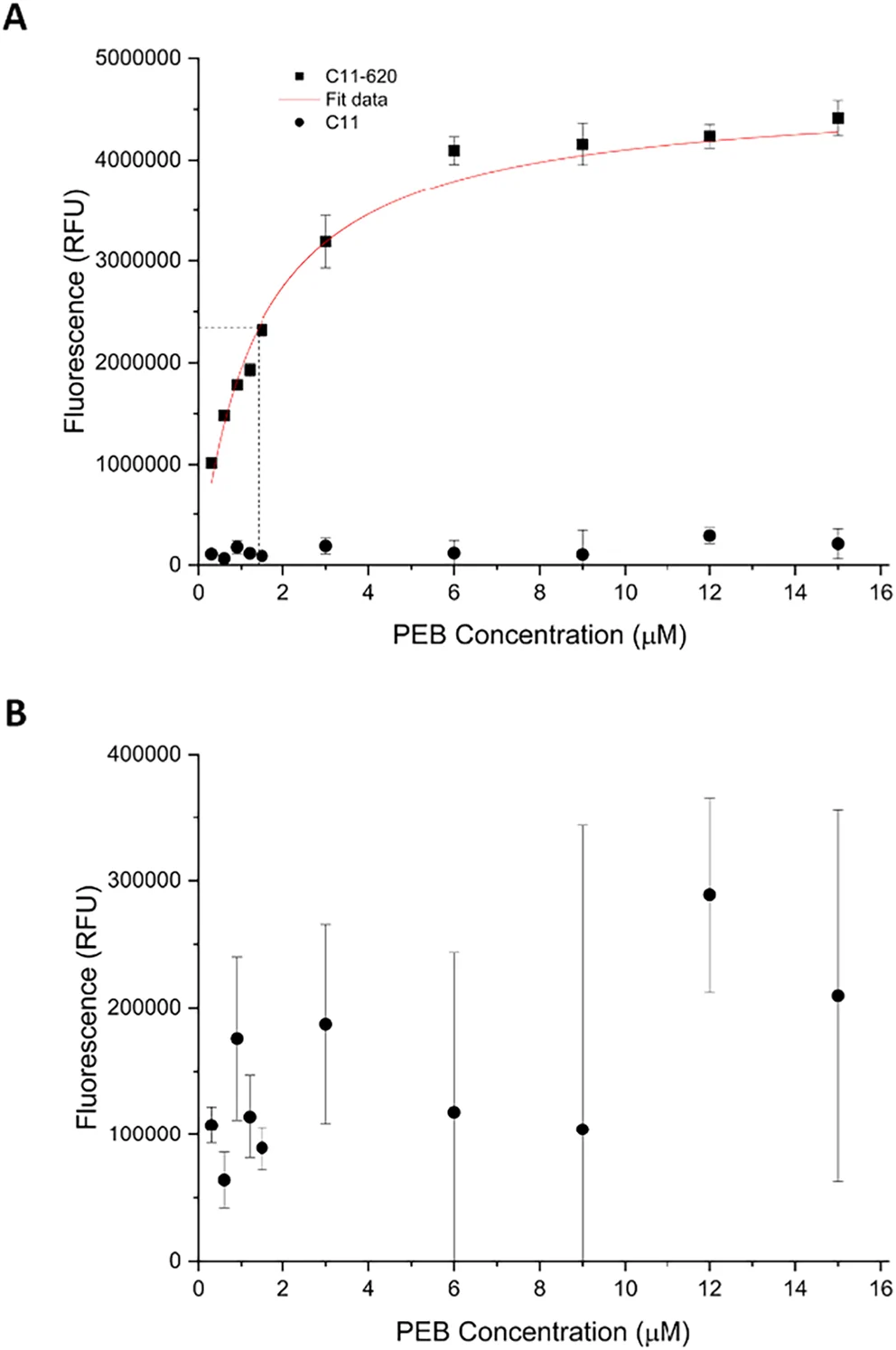
Measurements of PEB binding to C11 and C11-620 proteins. (A) PEB dose-dependent fluorescence emission for C11 (circles) and C11-620 (squares), excited at 525 nm, measured at 525 nm. C11-620 data fit using the one-site specific binding formula ((*B*_max_ × *x*)/(*K*_*d*_ + *x*)). (B) Zoomed graph of the C11 data collected as in panel (A). Error bars represent the standard error of the mean (*n* = 3).

**Figure 6:**
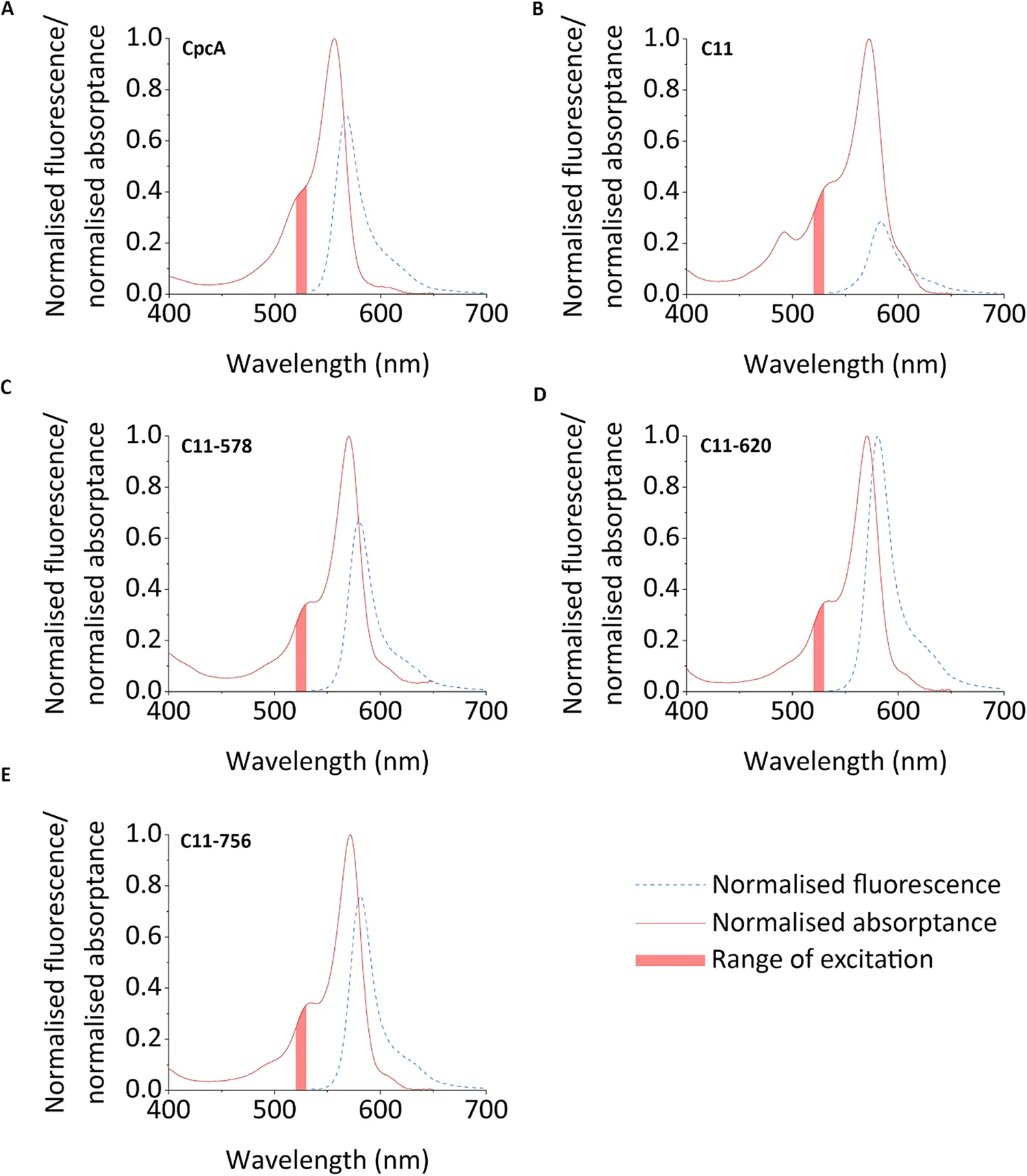
Fluorescence emission analysis of CpcA, C11 and the C11 redesigns. (A) CpcA, (B) C11 design, (C) C11-578 redesign, (D) C11-620 redesign, and (E) C11-756 redesign. The maximum absorptance (red outline) was normalized to 1 for each sample, and the fluorescence emission (dashed blue) spectra were scaled to illustrate their relative amplitudes. Excitation was from 520 to 530 nm and is indicated by the solid red bars. Fluorescence emission was measured from 535 to 700 nm. For calculations of relative and absolute fluorescence yields, see Table 1.

### Excited State Dynamics Revealed by Ultrafast Spectroscopy of Designed BPs

Transient absorption (TA) spectroscopy of CpcA, together with its fluorescence lifetime kinetics, has been detailed in a previous study by our group.^31^ In the present work, we focus on the ultrafast dynamics of C11 and its C11-620 descendant in buffered solution. Consistent with our earlier findings, the ultrafast TA spectra of CpcA (Figure 7A) are dominated by three principal features. The pronounced negative band centered at ∼560 nm is attributed to the overlap of ground-state bleach (GSB) and stimulated emission (SE), as highlighted by the blue-shaded region. Two positive excited-state absorption (ESA) bands are located below 500 nm and above 650 nm, respectively. The TA spectra of C11 (Figure 7B) exhibit analogous spectral characteristics, albeit with a slight redshift (∼15 nm) in the GSB/SE region and a markedly weak ESA band beyond 660 nm; this behavior is consistent with the red-shifted GSB and emission bands. The TA spectra of C11-620 (Figure 7C) are nearly identical to C11, except for the higher amplitude of the TA signals after 6 ns, which suggest a long-lived excited state potentially associated with triplets.

**Figure 7:**
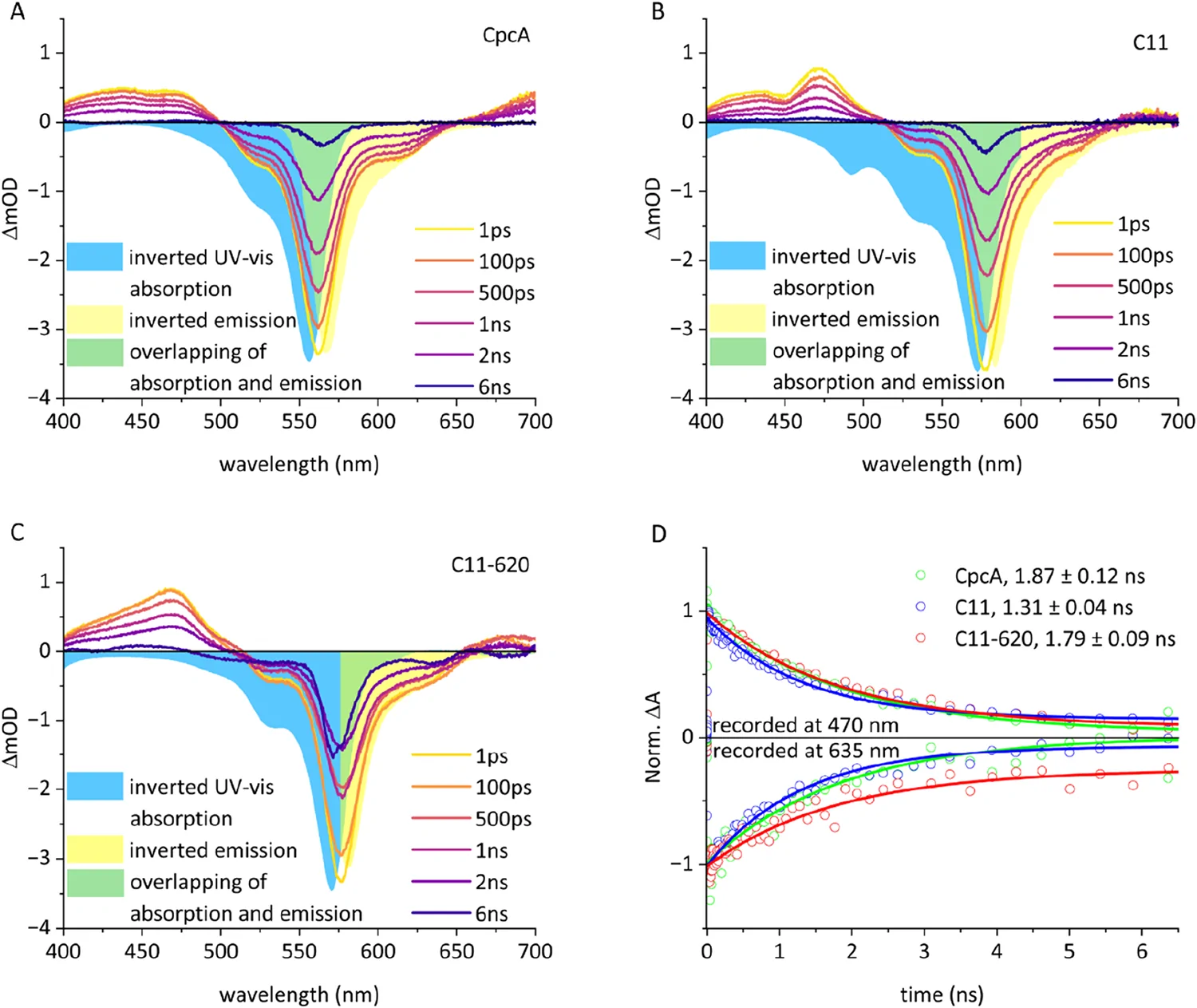
Transient absorption spectra at selected decay times for biliproteins. (A) CpcA, (B) C11, and (C) C11-620. The spectra of CpcA were recorded with 565 nm excitation, while those for C11 and C11-620 were obtained with 572 nm excitation. The inverted UV–vis absorption and emission spectra, taken from Figure 6, were scaled to match the amplitude of the negative absorption band. (D) Comparison of the transient absorption kinetics at 470 and 635 nm for CpcA, C11, and C11-620. Δ*A* is the difference between ground state absorption and the excited state at each time constant.

Monoexponential decay fits were applied to the transient absorption kinetics at 470 and 635 nm for CpcA, C11, and C11-620 (Figure 7D). CpcA exhibited a decay time of 1.87 ns, assigned to the S_1_ → S_0_ transition,^31^ with a similar value of ∼1.79 ns measured for C11-620. A faster decay of ∼1.31 ns was obtained for C11. The same trend was observed when the fluorescence lifetimes were recorded (Figure S10). The CpcA fluorescence lifetime has been measured previously, with a decay of 1.9 ns,^31^ comparable with C11-620 (2.04 ns), C11-578 (1.83 ns), and C11-756 (2.30 ns); all of these fluorescence lifetimes are much longer than the 0.74 ns measured for C11 (Figure S10).

The C11 fluorescence lifetime of 0.74 ns, measured with 485 nm excitation, was significantly shorter than the ∼1.31 ns decay time observed from TA measurements with 572 nm excitation. This discrepancy might be the result of the presence of multiple, spectroscopically distinct subpopulations in C11, as also indicated by the mismatch between the steady-state absorption and fluorescence excitation spectra (Figure S9). In particular, the excitation peak below 500 nm for C11 was absent when detecting emission at 630 nm (Figure S9A); therefore, fluorescence measurements with 485 nm excitation include <500 nm excitation features with shorter lifetimes, whereas TA excludes them. The major difference in the TA measurements between CpcA, C11 and the C11 descendants, C11-578, C11-756 (Figure S10A,B), and C11-620 (Figure 7C,D), is a long-lived excited state at 460 nm, and negative absorption features below 550 nm and at ∼670 nm, both after 6 ns in C11-620. The nature of these long-lived signals is currently unknown.

### Structural Modeling of C11 and Redesigned BPs

Each design was relaxed using Rosetta, and the highest scoring structure from the ten generated was selected for analysis. The structural models for C11 and the redesigns are displayed in Figure 8; the insets show the residues predicted to be involved in hydrogen bonding and other interactions with the bilin, as well as the cysteine that forms the thioether linker. The insets also highlight the similar bilin conformations, indicating very little change in the coplanarity of the A- and D-rings, consistent with the similar absorption spectra for C11 and the new designs.^38^ In the original C11 structure, C11-578 and C11-756 the bilin is predicted to have two hydrogen bonds, but there are three for C11-620. These can be seen in Figure 8 and an enlarged version is highlighted for C11-620 and C11 in Figure S13; these interactions are also summarized in Table S2. Although the extinction coefficients of C11-620 and C11-756 are comparable (Table 1), C11-620 surpasses C11-756 in terms of brightness by ∼46%, due its high fluorescence yield, possibly due to a combination of the smaller SASA and additional predicted hydrogen bond from Arg-20. The charge density surrounding the D-ring displays no correlation with the minor shifts observed for the wavelength maxima of the bilin in the new C11 designs (data not shown). This suggests that for these BPs it is the hydrogen bonds, from charged residues, rather than the charges themselves, that contribute to the charge-associated red/blue shifting of the bilin absorption maxima. Efforts to design in hydrogen bonds from a range of charged and other donor types, could provide insights into this issue.

**Figure 8:**
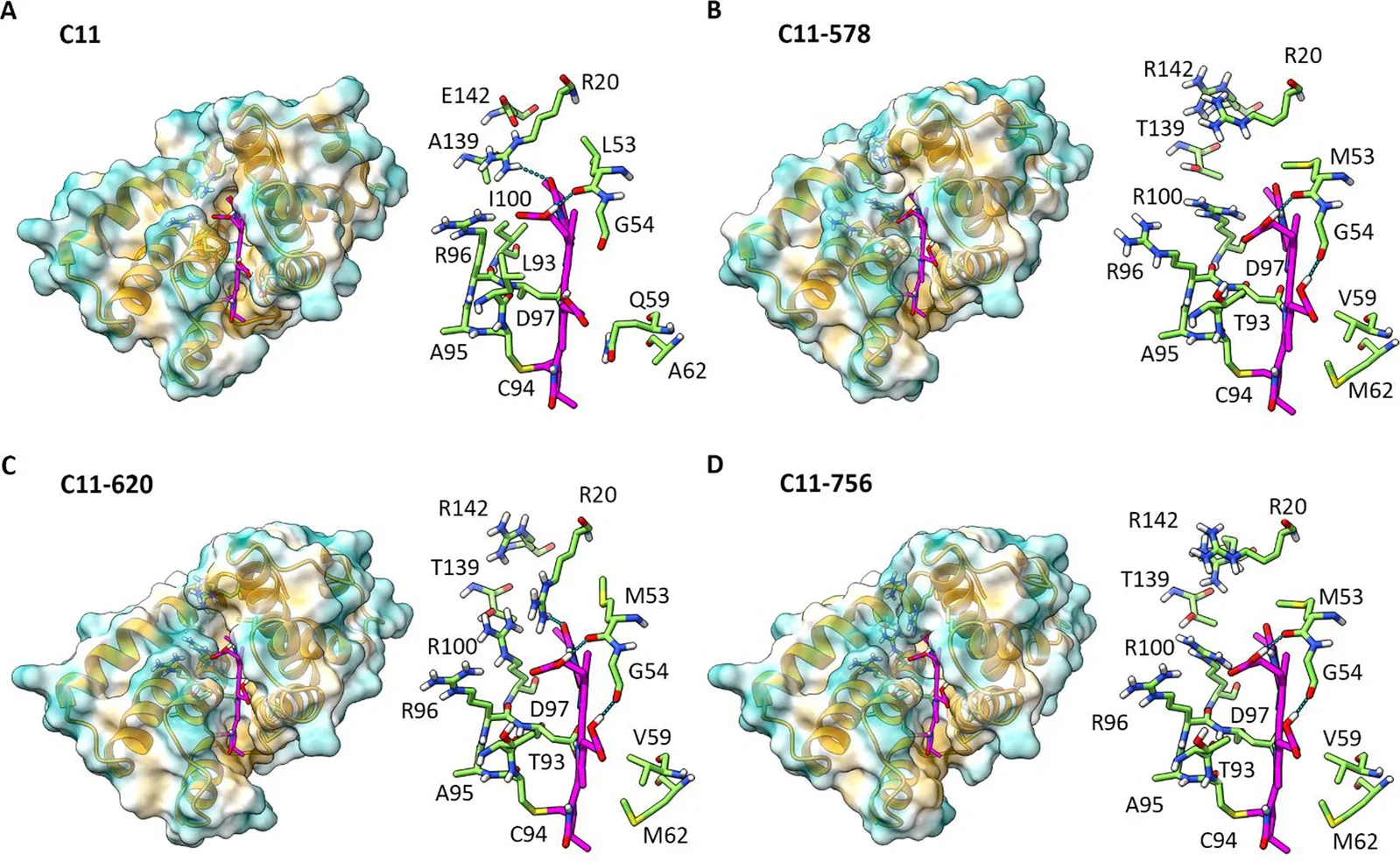
Rosetta relaxed structures of biliproteins. (A) C11, (B) C11-578, (C) C11-620, (D) C11-756. The respective bilins and interacting residues are shown to the right of each model. All bilins are attached via a thioether linkage to cysteine-94. Hydrogen bonds are shown in cyan dotted lines. Residues within 5 Å of PEB that have been modified from the original C11 design are displayed, as are residues 94–97 that comprise the “CARD” motif. The surface colors (gold–hydrophobic, blue–hydrophilic) provide an indication of the hydrophobicity of the proteins.

In order to investigate possible contributions from factors other than hydrogen bonding to the enhanced performance of C11-620, we analyzed the predicted BP structures using the Rosetta scoring function, given that LigandMPNN currently lacks the functionality of scoring a given ligand-protein complex. The score associated with the ligand itself was improved by −3.7 Rosetta Energy Units (REU) (see Table S4; column “total_score”), which primarily is accounted for by more favorable Lennard–Jones attraction, Lazaridis–Karplus solvation energy, Coulombic electrostatic interaction, and hydrogen bonds with the protein (see Tables S4 and S5). Additionally, the score associated with the global protein fold is improved by −55.1 REU (see Table S5 column “total_score”), which is associated with energy terms similar to those for the ligand. However, the Dunbrack rotamers are also more favorable and there is less Lennard-Jones repulsion in C11-620 (see Tables S4 and S5). Interestingly, AF3 scores for the interface predicted template modeling (iPTM) between the ligand and the backbone are slightly worse for C11-620 (0.89) than C11 (0.92), suggesting this is not a suitable metric for tightness of binding. The same is observed for the ligand predicted local distance difference tests (pLDDTs), which are 89.7 and 88.6 for C11 and C11-620, respectively. There are however modest improvements of the protein backbone pLDDTs, which are 88.0 and 93.8 for C11 and C11-C620 (Figures S11C–D and S12), respectively, which suggests some of the improved optical properties may come from a more rigid backbone. Interestingly, the original C11 is predicted to be more unstable in the absence of the ligand (Figures S11E,F and S12), which suggests that, unlike C11-620, much of the stability observed in the C11 holoprotein comes from ligand-protein interactions. Given the flexible nature of the ligand, this may dissipate bilin excited states through motions of ligand and protein. AF3 models, along with their pLDDT, predicted aligned error (PAE) and iPTM scores, are shown in Figures S11 and S12.

Redesign of C11 to form C11-620 was accompanied by drastic mutations, such as K to D, that are frequently associated with a “co-evolution” of a nearby residue. For example, C11 residues A67 and F71 “co-evolve” to F67 and A71 in C11-620. Similarly, C11 residues S31-E33 become D31-S33 in C11-620, and K19-E142 change to D19-R142 (Figure S11A,B). Other alterations going from C11 to C11-620 such as I110R and R89L are located around the chromophore pocket, and so are likely involved in tighter PEB binding. Such changes in the primary sequence are accommodated by the tertiary structure, thereby maintaining similar packing (Figure S11).

## Discussion

The extinction coefficient and the fluorescence yield of bilins are intrinsically coupled to the constraints imposed by protein binding, so bilins can act as optical reporters for screening and testing designs for binding small molecules. These properties of the PEB bilin were used successfully to evaluate the capacity of RFDiffusionAA to design small molecule binding proteins.^25^ De novo BP designs have further uses as potential components of reimagined light-harvesting assemblies in photosynthesis; however, in such systems they would have to match the performance of native BPs. We tested two of the de novo designs that emerged from the original small molecule binding screen, C11 and F9, by comparing their stability and optical properties with a native BP, CpcA-PEB, a component of the PBS. This analysis showed that C11 and F9 were unsuitable as light-harvesting components, so we focused on C11 and used a computational pipeline to iteratively refine and improve the protein and its PEB binding site. The C11 descendants from this pipeline, C11-578, C11-620 and C11-756 exhibited significantly improved stability, extinction coefficients and fluorescence quantum yields, and structural modeling shows the basis for enhancing the constraints on PEB within the binding pocket. The improvement achieved through a second design iteration raises an interesting question regarding the initial design–is it possible to achieve C11-620-like designs in a one-shot design process? Possibly so, but this could be very expensive computationally, and the two-stage process used here involving an initial screen followed by a second redesign step has produced functionally useful outputs for this model bilin system without having to screen a large number of candidate designs.

Our simple computational pipeline works by optimizing the ligand confidence metric output from a LigandMPNN calculation. It also leverages the power of Rosetta to generate new input structures for LigandMPNN without the GPU-costly folding of all output LigandMPNN sequences with AF3, resulting in a design pipeline that can be run in only a few hours from start to finish, accessing only the freely available GPUs on the AF3 server. A similar pipeline could be applied to other small molecule binders, but recent work is already advancing this rapidly developing field. The present study predates the release of the AF3 code on GitHub, which facilitates the generation of protein–ligand structures and therefore simplifies the task of generating the next input structure for LigandMPNN without the need for Rosetta Relax. It also provides metrics (iPTM) on the confidence of ligand-protein interaction which could benefit selection of sequences. There are likely hidden complexities involved in ligand binding, although the advent of protein–ligand structure prediction has simplified the design process. Boltz, a tool for predicting biomolecular interactions based on the affinity and probability of ligand binding, allows users to sift through designs that have high ligand confidences to make a more refined selection.^39^ Reproductions of AF3 by other groups are leading to competitive advancements in the field of protein–ligand structure prediction, such as Protenix.^40^ Through the use of RFDiffusionAA,^25^ LigandMPNN^33^ and protein–ligand structure prediction, the field of ligand-binding protein design is becoming commonplace. The difficult next steps will be the introduction of acceptable levels of functionality to designs, particularly for enzymes and large-scale assemblies.

The absorption, TA and fluorescence experiments show that the redesign pipeline has improved the functionality of the original C11 BP, placing the C11-620 redesign within the working parameters of the CpcA PBS component in terms of excited states and fluorescence lifetimes, making it an ideal candidate for the generation of phycobilisome-like oligomers that can be utilized for light harvesting. These fluorescence lifetime values are also comparable to the excited state lifetime of the assembled PBS, which has been measured to be ∼1.7 ns.^41^ The fluorescence lifetime is an important characteristic for a light-harvesting antenna, so interchromophore energy transfer rates will be an important consideration for de novo BPs engineered to form higher order assemblies. In the native phycobilisome of *Synechocystis* sp. PCC 6803, energy transfer rates are on the order of 1–10 ps^–1^,^10^ which is a useful guide for assessing the performance of designed BP oligomers.

The method used to enhance the properties of C11 to form C11-620 can be applied to the red-shifted F9 BP (Figure 1), which is also in need of improvement. Starting with C11-620, and combining with F9 and their descendants, energy cascades can be created from conjoined BPs that form oligomers, generating new BP assemblies. Structural studies of PBSs illustrate the range of possibilities created by the natural variety of bilin-binding sites and the way that individual phycobiliproteins are deployed within overall PBS architecture.^4^ The present study provides a computationally low-cost approach for improving designs for binding small molecules, exemplified here by the rational redesign of bilin-binding sites, and it forms the basis for further synthetic biology studies of photosynthesis. Such work includes the de novo design of phycobiliprotein oligomers, generating a fresh set of options for constructing membrane-extrinsic light-harvesting assemblies.

## Conclusions

Here, we present an approach for improving proteins designed to bind small molecules, exemplified in our study by the PEB ligand. The absorption and fluorescence properties of bilins such as PEB act as reporters of the fit within the protein binding site, and our analyses showed that one of our original designs, C11, required improvement. We devised a computationally efficient pipeline to redesign C11, generating C11 variants with enhanced stability, absorption properties and fluorescence yields with an up to 13-fold improvement in overall brightness. We conclude that de novo proteins that emerge from initial screens can be improved by computational redesign, and that bilins are well suited to evaluate protocols for enhancing the binding of small molecules. The optical properties of bilins, tightly bound within redesigned de novo proteins, have potential for fluorescence imaging, optogenetics, and biosensing and for constructing oligomeric, membrane-extrinsic assemblies that harvest light for photosynthesis.

## Supplementary Material



## Notes

Protein sequences; absorption spectra; size exclusion chromatography; ligand confidence scores; fluorescence excitation and absorptance spectra; transient absorption spectra; AF3 and Rosetta models; PAE and iPTM plots; flowchart of the redesign pipeline; deconvoluted native mass spectra; theoretical and observed molecular weights; hydrogen bond donors to PEB, and Rosetta scores (PDF)

## #

Department of Biochemistry, University of Washington, Seattle, Washington 98195, United States and Paul G. Allen School of Computer Science and Engineering, University of Washington, Seattle, Washington 98195, United States

## ∇

Department of Biochemistry, University of Washington, Seattle, Washington 98195, United States and Howard Hughes Medical Institute, University of Washington, Seattle, Washington 98105, United States

## ○

School of Mathematical and Physical Sciences, University of Sheffield, Brook Hill, Sheffield S3 7HF, U.K.

## ◆

Plants, Photosynthesis, and Soil, School of Biosciences, University of Sheffield, Firth Court, Western Bank, Sheffield S10 2TN, U.K.

## Abbreviations

PEB: phycoerythrobilin
PCB: phycocyanobilin
PΦB: phytochromobilin
PUB: phycourobilin
PVB: phycoviolobilin
RFDiffusionAA: RosettaFold Diffusion All-atom
REU: Rosetta energy units
AF3: AlphaFold 3
FWHM: full width at half-maximum
TEV: Tobacco Etch Virus
FL: fluorescence
SASA: solvent-accessible surface area
IMAC: immobilized metal affinity chromatography
TA: transient absorption
BP: Biliprotein
GPU: Graphics Processing Unit
iPTM: interface predicted TM-score
*E. coli*: *Escherichia coli*
CMOS sensor: complementary metal-oxide-semiconductor sensor
SEC: size exclusion chromatography
DTT: dithiothreitol
