# Supplementary material for: Improved Stability and Brightness Following Iterative Redesign of a De Novo Biliprotein
Source: Biochemistry. 2026 Jun 15;65(16):2566–78. doi: 10.1021/acs.biochem.6c00166 (PMC13492287; doi:10.1021/acs.biochem.6c00166)
Supplement: Supplementary file 1 [file bi6c00166_si_001.pdf]

Supporting Information

Improved Stability and Brightness Following Iterative Redesign of a De Novo Biliprotein

Felix S. Morey-Burrows[1], Tingxiang Yang[1], Woody Ahern[2], David Baker[2], Andrew Hitchcock[1], Graham J. Leggett[3], Jenny Clark[4], Aneika C. Leney[5], and C. Neil Hunter[1]**

[1]School of Biosciences, University of Sheffield, Firth Court, Sheffield, S10 2TN, UK.
[2]Institute for Protein Design, University of Washington, Seattle, WA 98105, USA.
[3]Department of Chemistry, University of Sheffield, Sheffield, UK, S3 7HF, UK.
[4]School of Mathematical and Physical Sciences, University of Sheffield, Hicks Building, Hounsfield Road, Sheffield, S3 7RH, UK.
[5]School of Biosciences, University of Birmingham, Birmingham, B15 2TT, UK.

*To whom correspondence should be addressed: c.n.hunter@sheffield.ac.uk, a.leney@bham.ac.uk

Contents

S2. Fig. S1. Protein sequences for native CpcA, and designed C11 and F9 biliproteins.
S3. Fig. S2. Absorption spectra of native CpcA, and designed C11 and F9 biliproteins from initial screen.
S4. Fig. S3. Size exclusion chromatography of native CpcA, and designed C11 and F9 biliproteins.
S5. Fig. S4. Ligand confidence scores for a series of protein design iterations.
S6. Fig. S5. Size exclusion chromatography of the designed C11 and new C11 variant biliproteins.
S7. Fig. S6. Normalised absorption spectra of C11 and the new C11 variants.
S8. Fig S7. Absorption spectra of IMAC-purified cysteine mutants of CpcA and C11-620.
S9. Fig S8. Fluorescence excitation and absorptance spectra of C11 redesigns relative to the original C11 design and CpcA.
S10. Fig. S9. Absorption spectra illustrating the stability of C11 redesigns over 24 h storage at 4°C relative to the original C11 design.
S11. Fig. S10. TA spectra at selected decay times and fluorescence kinetics of C11 and its descendants, C11-578, C11-620, and C11-756.
S12. Fig. S11. AF3 and Rosetta models highlighting the major differences between the original C11 design and the C11-620 redesign.
S13. Fig. S12. PAE and iPTM plots for the apo- and holo- C11 and C11-620 AF3 models.
S14. Fig. S13. Zoomed models of C11 (A-B) and C11-620 (C-D) highlighting the PEB-protein interactions and the CARD motif (residues 94-97).
S15. Fig. S14. Flowchart of the pipeline applied to the redesign of the C11 protein.
S16. Fig. S15. Deconvoluted native mass spectra.
S17. Table S1: Theoretical and observed molecular weights for most abundant unmodified and modified protein species as determined by native mass spectrometry.
Table S2: Hydrogen bond donors to PEB in the original C11 and new designs.
S18. Table S3. Description of the scoring components for the ref2015 scoring function.
S19. Table S4. Rosetta Scores of the PEB ligand for C11 and C11-620. S20. Table S5. Rosetta Scores of the C11 and C11-620 proteins, with their PEB ligands.

CpcA

MSHHHHHHSGGSGSGENLYFQSGSGSMKTPLTEAVSTADSQGRFLSSTELQIAFGRLRQANAGLQAA
KALTDNAQSLVNGAAQAVYNKFPYTTQTQGNNFAADQRGKDKCARDIGYYLRIVTYCLVAGGTGPLDE
YLIAGIDEINRTFDLSPSWYVEALKYIKANHGLSGDARDEANSYLDYAINALSGS

C11

MSHHHHHHSGGSGSGENLYFQSGSGSVTEEDIKKRKKILLEVLKKRAEEAGIKLEFSDEVLNDISADLTP
AILRLLQALGASPEQAQALVSLAEGIFSGEIDLEALAEELLASGRVNELCARDLAIISLFVNPEEARELAERM
LTGLGYSEEEIEEFLKNVLESAEKEIEEARKKVGS

F9

MSHHHHHHSGGSGSGENLYFQSGSGSSEGQERLKEALTRLAEAYERLAEQLERVGLPGEPAYELAEEAR
ELAEEADTEFGSLEFVRRVAELLDRFAEQILDTVLALALSDPSLTPEQRAKLEALLAEAEAARAEVAALEAA
EPPDLCARDQAKLRMYEAYLKALEALPPFPDPDLQRVLLLTAEAAKLTLEALALAFEAAGLTEVAQAVRK
LLERAEKIVERVQAQGS

Fig. S1. Protein sequences of native CpcA, and designed C11 and F9 biliproteins.

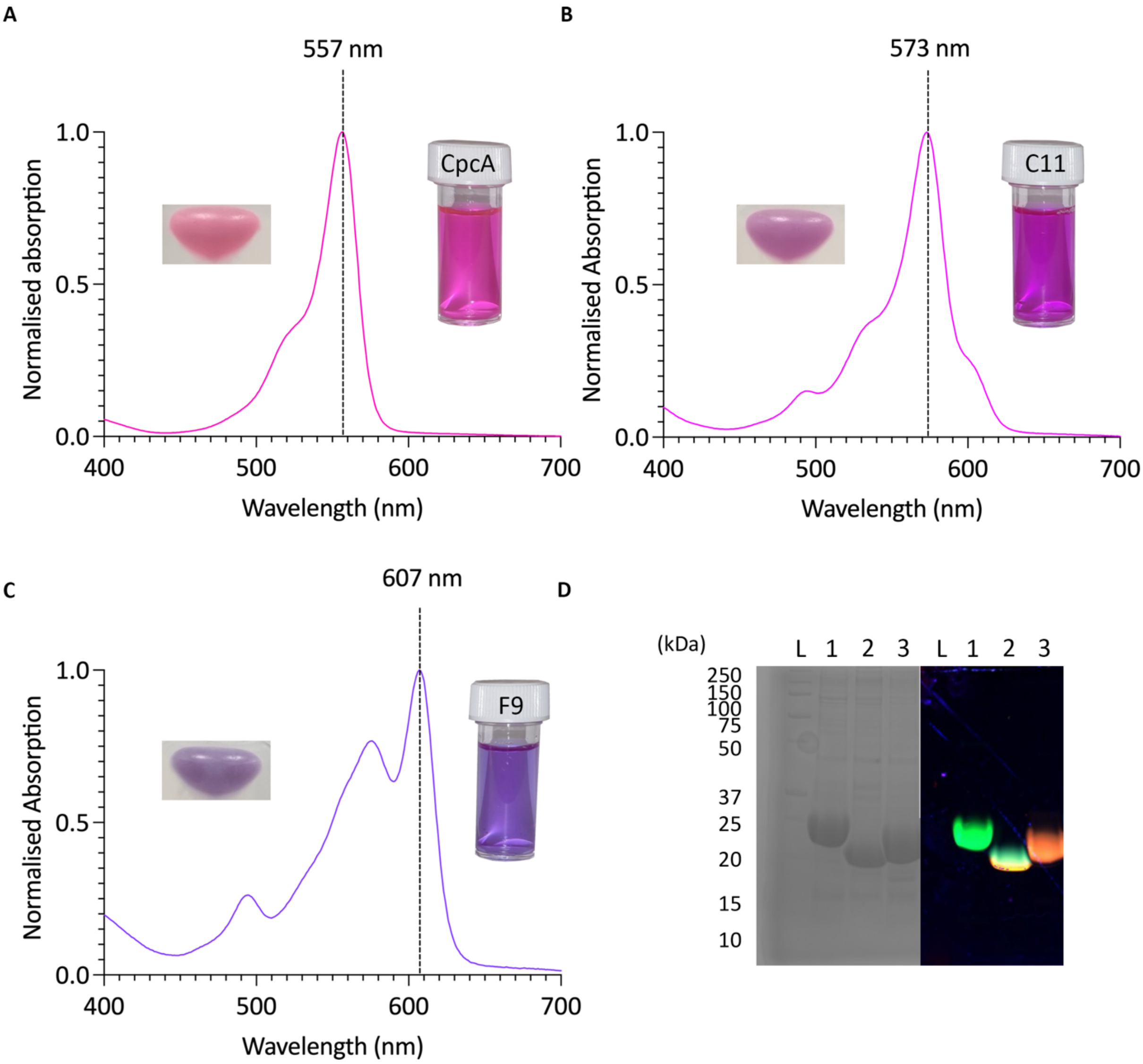

Fig. S2. (A-C) Absorption spectra of native CpcA, and designed C11 and F9 biliproteins from the initial screen. (D) SDS-PAGE of protein fractions eluted from IMAC of CpcA, C11 and F9 (lanes 1-3, respectively). L denotes the ladder of proteins with a range of molecular masses numbered in kDa. The left image is the Coomassie stained gel; the right image is the same gel but imaged by detecting the fluorescence of protein-attached bilins, using Cy3 (green signal) and Cy5 (red signal) filters.

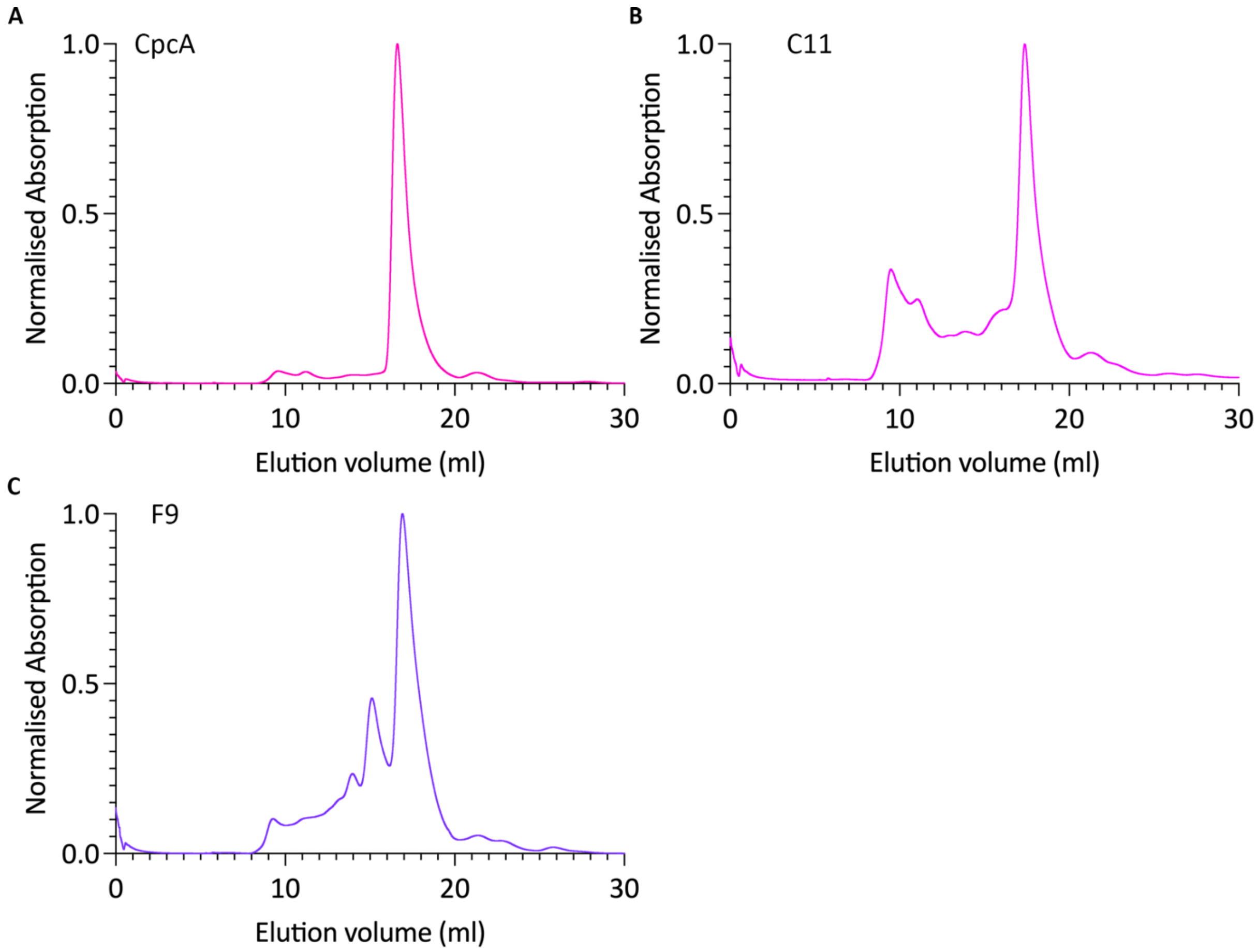

Fig. S3. Size exclusion chromatography of native CpcA, and designed C11 and F9 biliproteins, monitored at 280 nm.

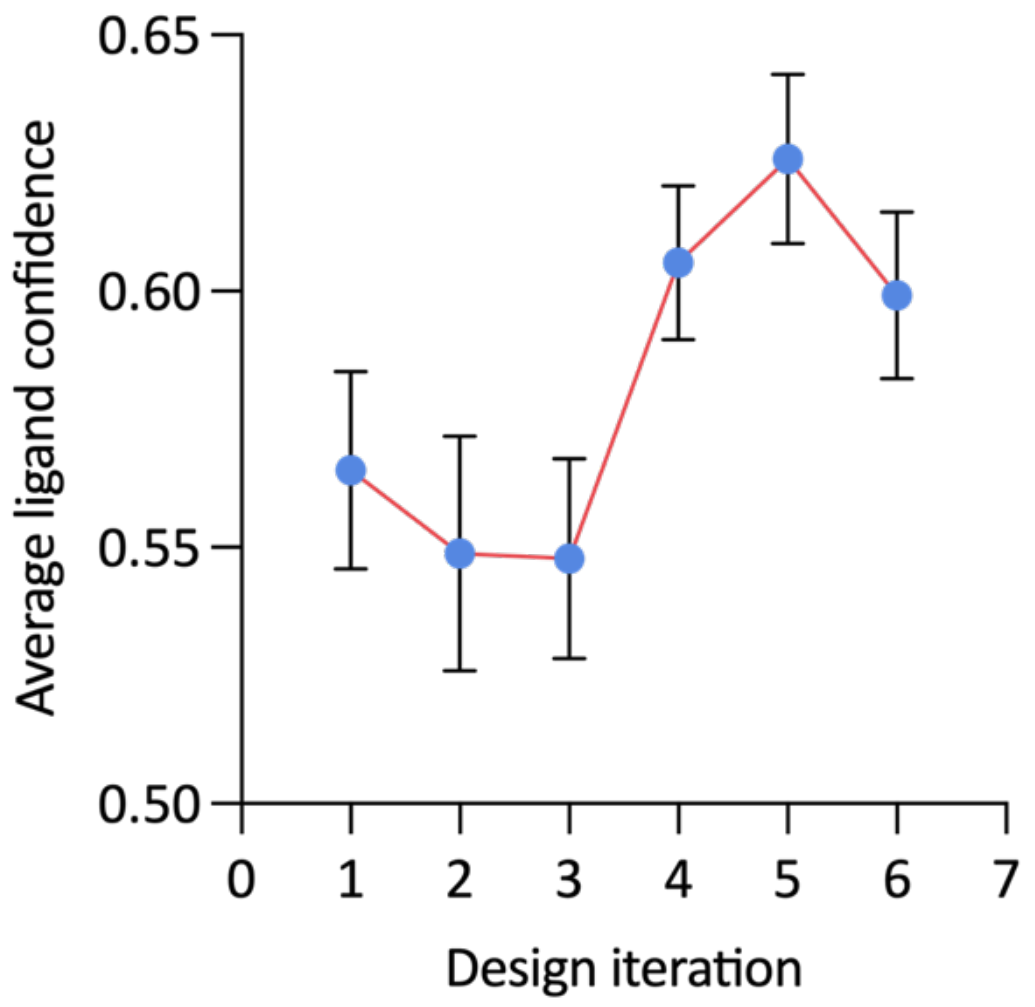

Fig. S4. Ligand confidence scores for a series of protein design iterations.

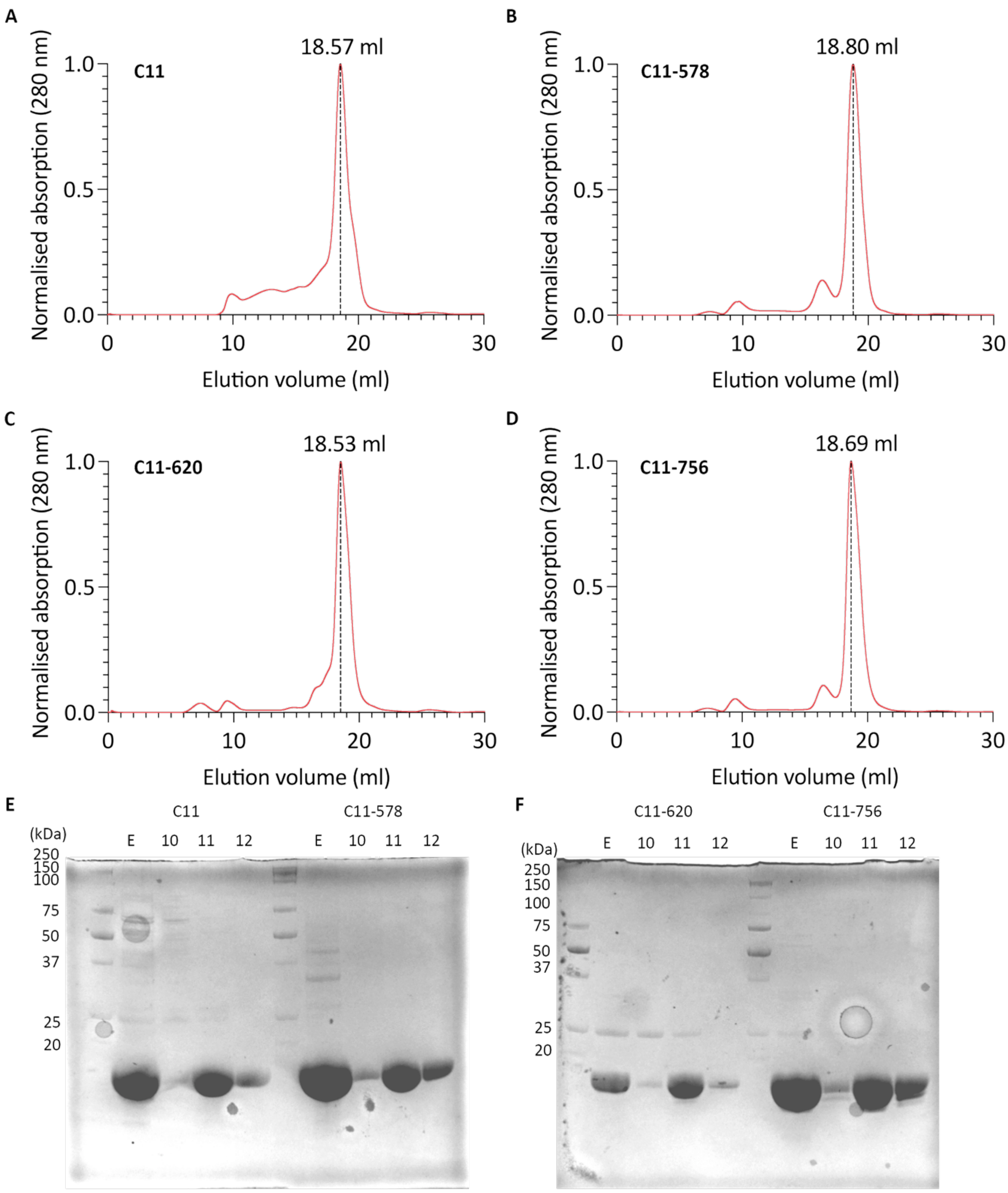

Fig. S5. (A-D) Size-exclusion chromatography of the designed C11 and new C11 variant biliproteins, monitored at 280 nm. (E-F) SDS gel electrophoresis of fractions eluted from the size exclusion columns. Lanes E are the proteins eluted from the initial IMAC column, and 10-12 are the numbers for fractions eluted from the subsequent size-exclusion column.

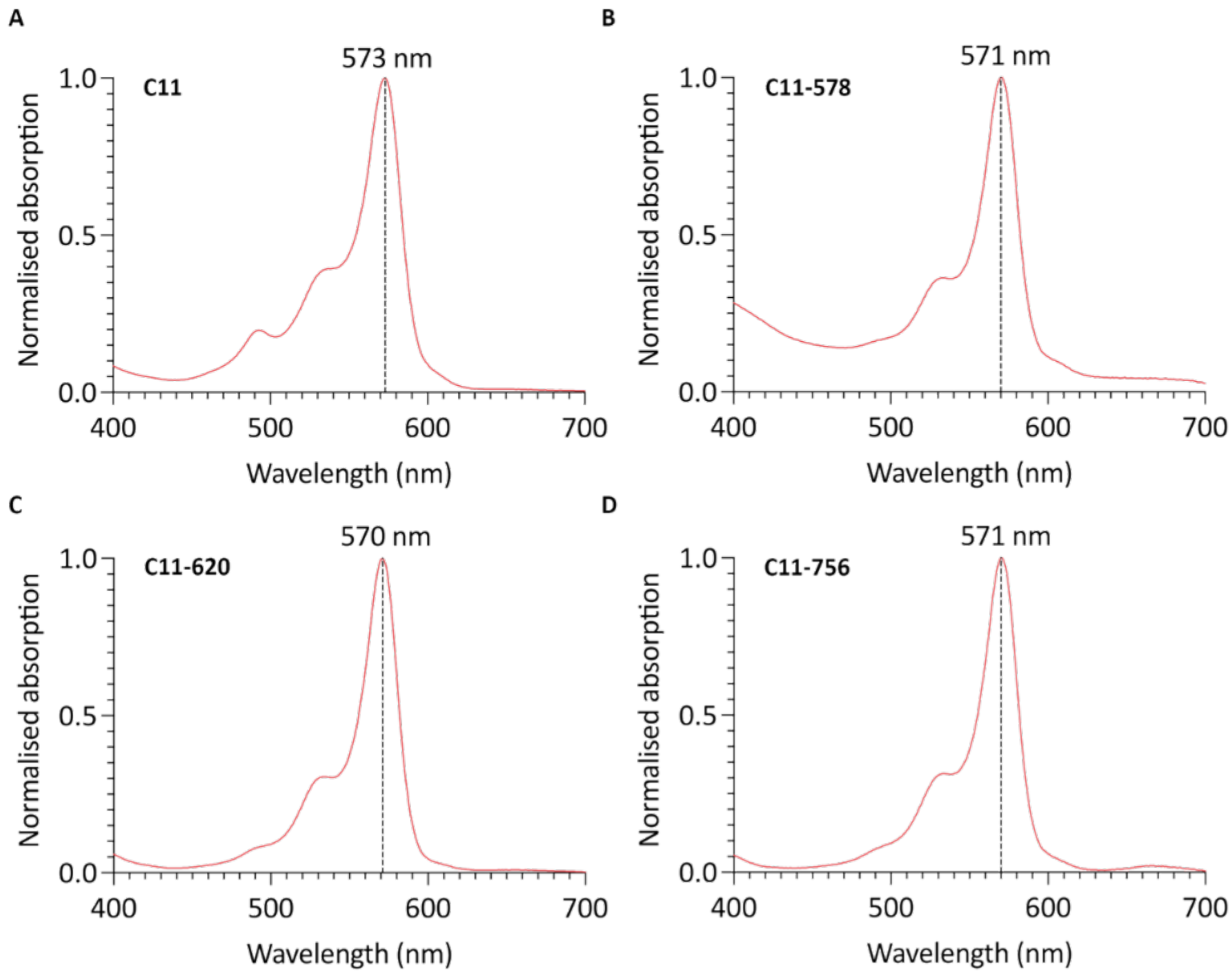

Fig. S6. Normalised absorption spectra of C11 and the new C11 variants.

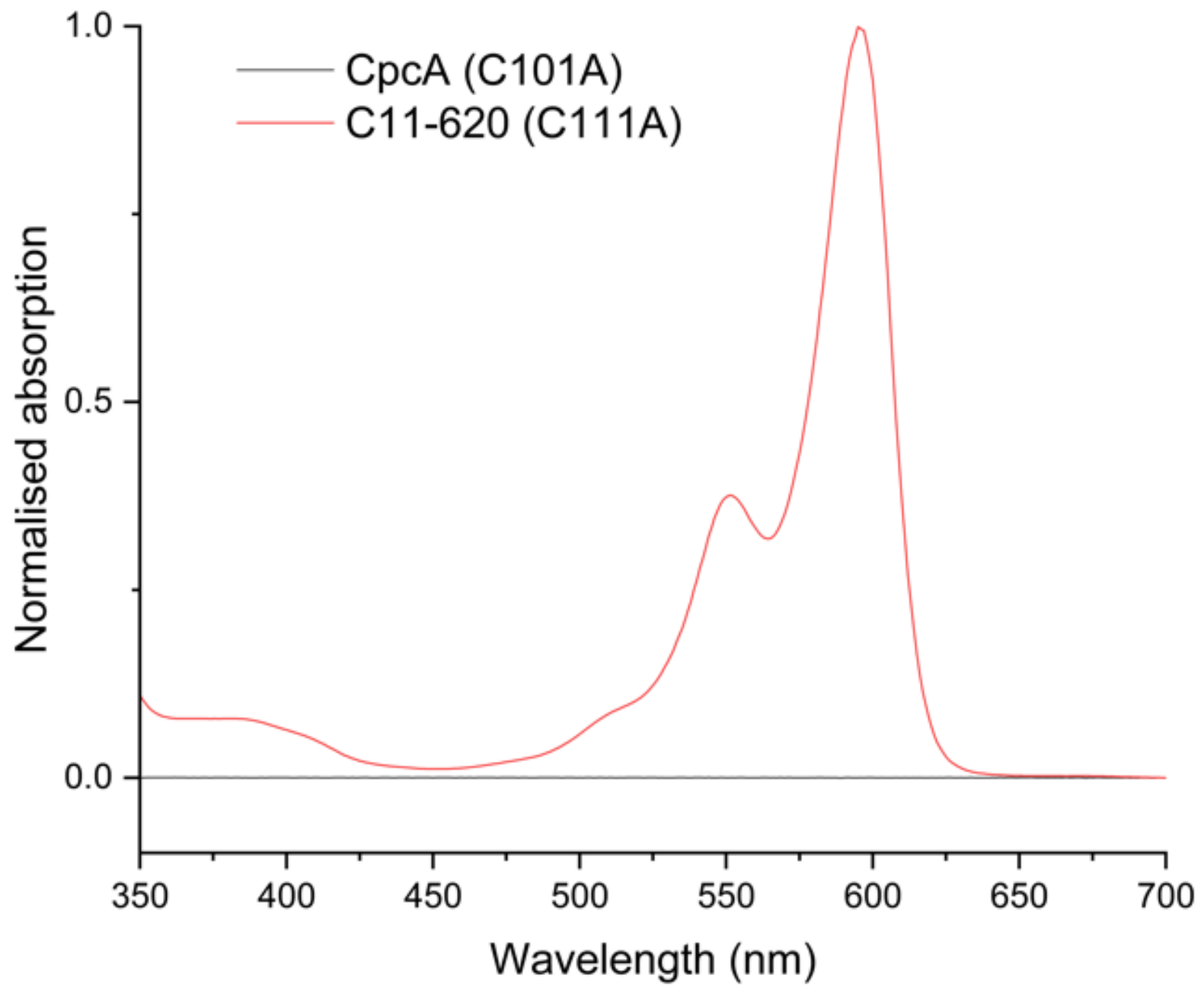

Fig S7. Absorption spectra of IMAC-purified cysteine mutants of CpcA (black) and C11-620 (red).

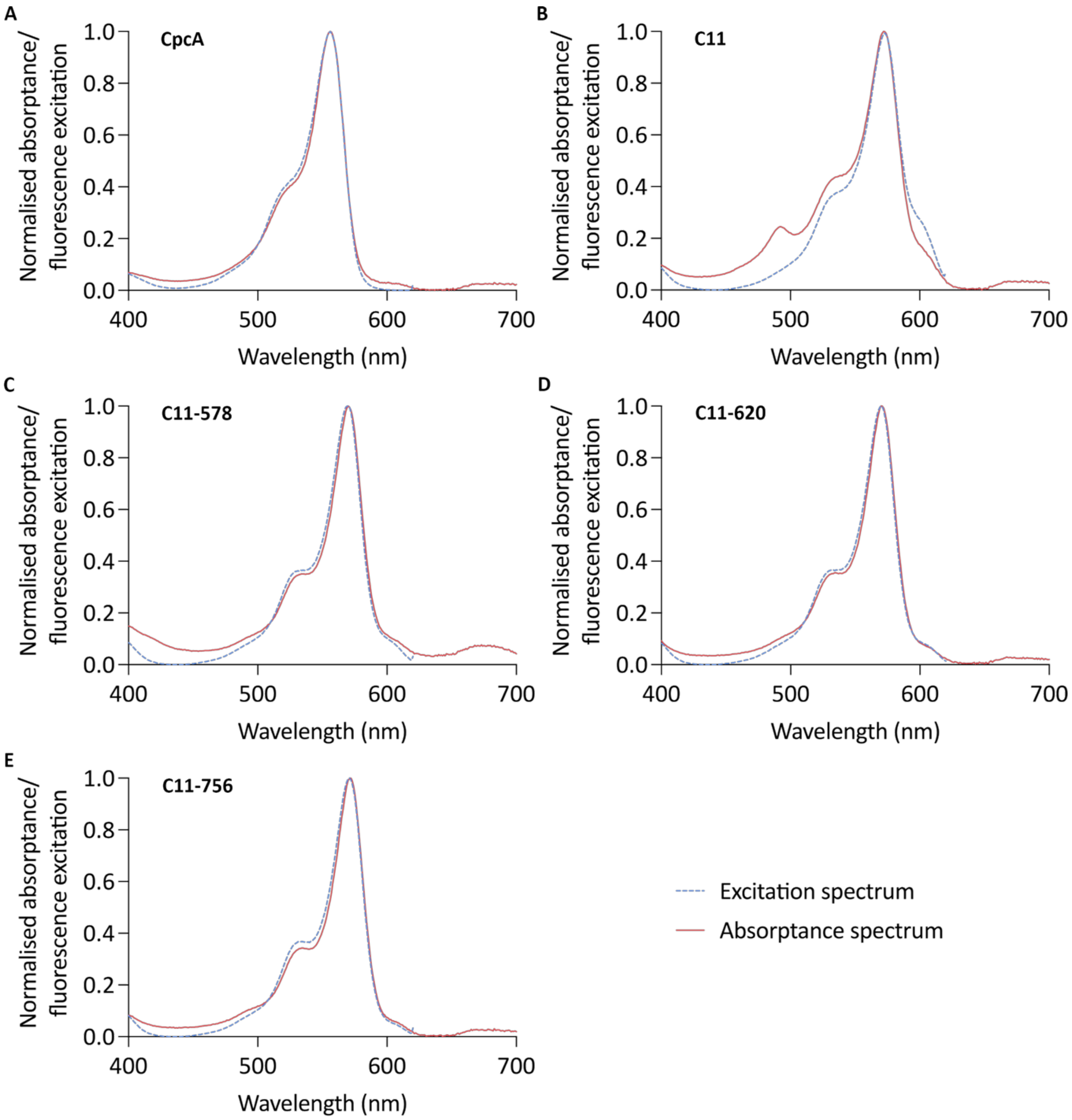

Fig S8. Fluorescence excitation (dashed blue line) and absorptance (solid red line) spectra of C11 redesigns relative to the original C11 design and CpcA.

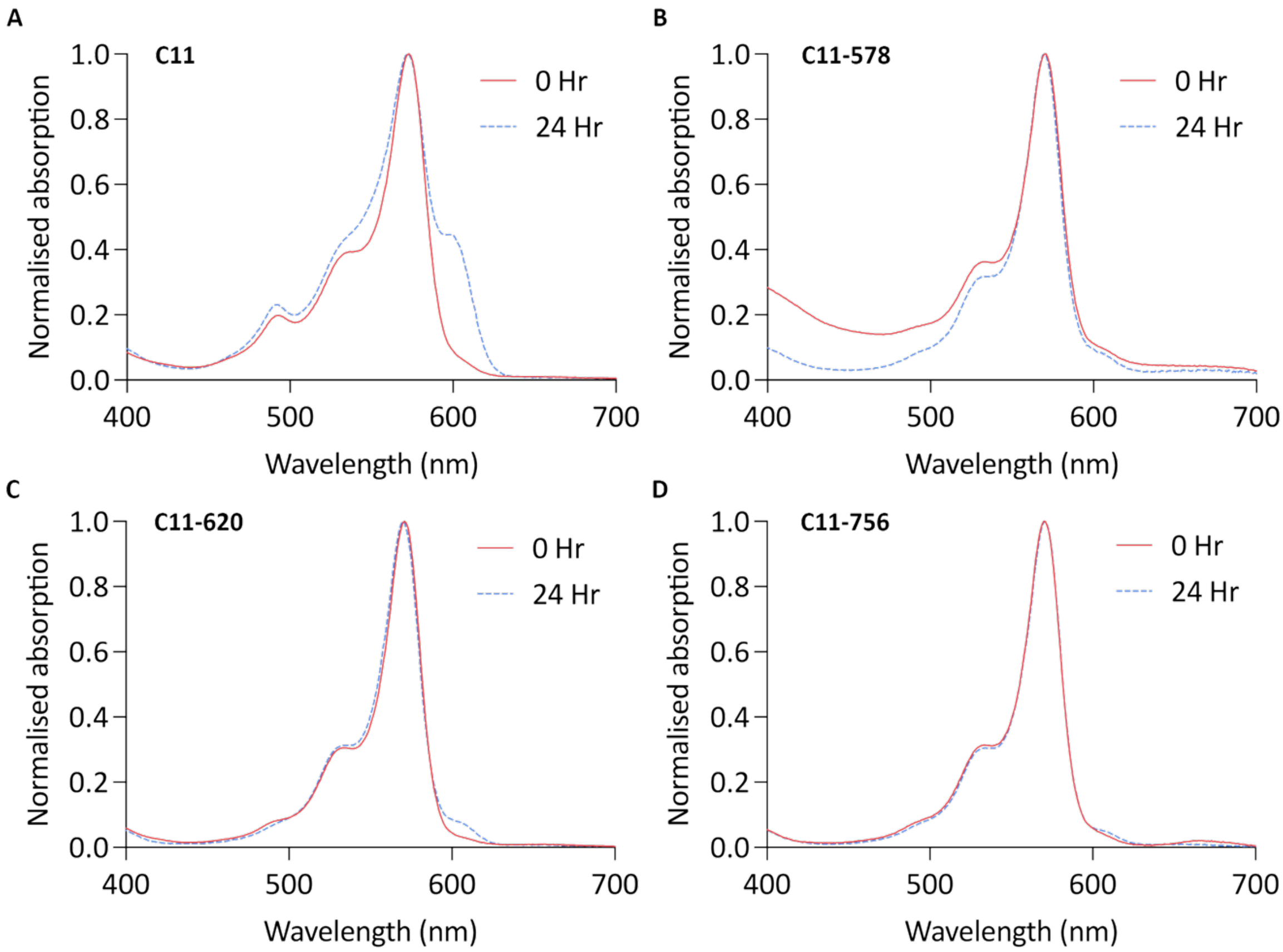

Fig. S9. Absorption spectra illustrating the stability of C11 redesigns over 24 h storage at 4°C relative to the original C11 design.

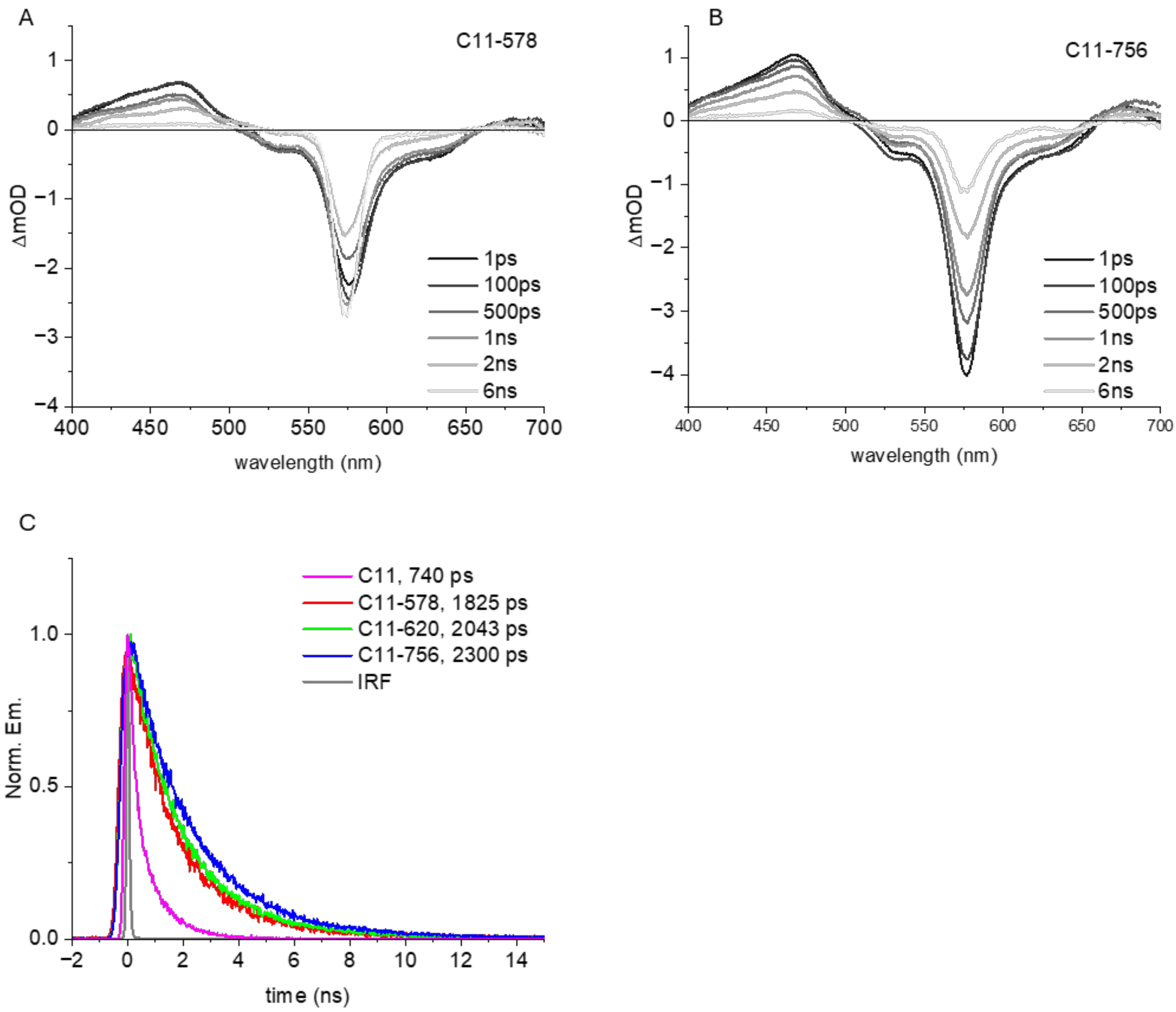

Fig. S10. TA spectra at selected decay times for (A) C11-578, (B) C11-756, and (C) fluorescence kinetics of C11 and its descendants, C11-578, C11-620, and C11-756.

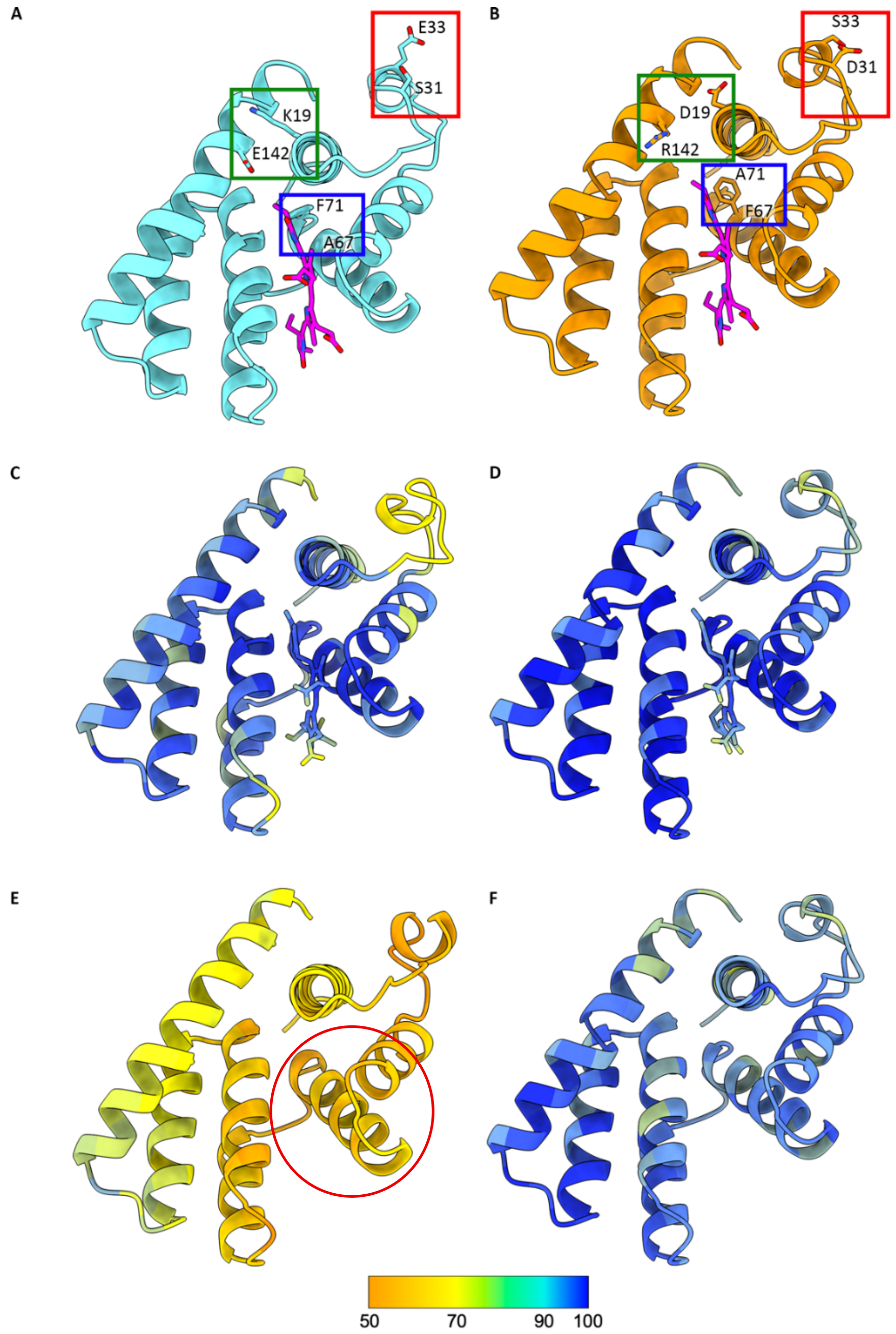

Fig. S11. AF3 and Rosetta models highlighting the major differences between the original C11 design (A,C,E) and the C11-620 redesign (B,D,F). (A-B) Rosetta models displaying non-conserved residues between the two proteins found within 5 Å of the ligand. The boxes highlight changes which appear to be non-conserved but have "co-evolved" with another residue. In the blue boxes in (A, B), the C11 residues A67 and F71 "co-evolve" to F67 and A71 in C11-620. Similarly, S31 and E33 change to D31 and S33 (red boxes) and E142 and K19 change to R142 and D19 (green boxes). (C, D) AF3 models of the proteins, each with a bound bilin, where both C11 (C) and C11-620 (D) show comparable average pLDDTs with C11-620 being slightly higher (C11 = 87.8; C11-620 = 93.1). (E, F) AF3 models of the apo-proteins, where large differences between the C11 (E) and C11-620 (F) average pLDDTs are observed (C11 = 61.4; C11-620 = 89.5). The red ellipse highlights a helix with the majority of non-conservative changes, corresponding to residues ~60-70 facing the bilin ligand. The scale bar represents the per residue pLDDTs of the models.

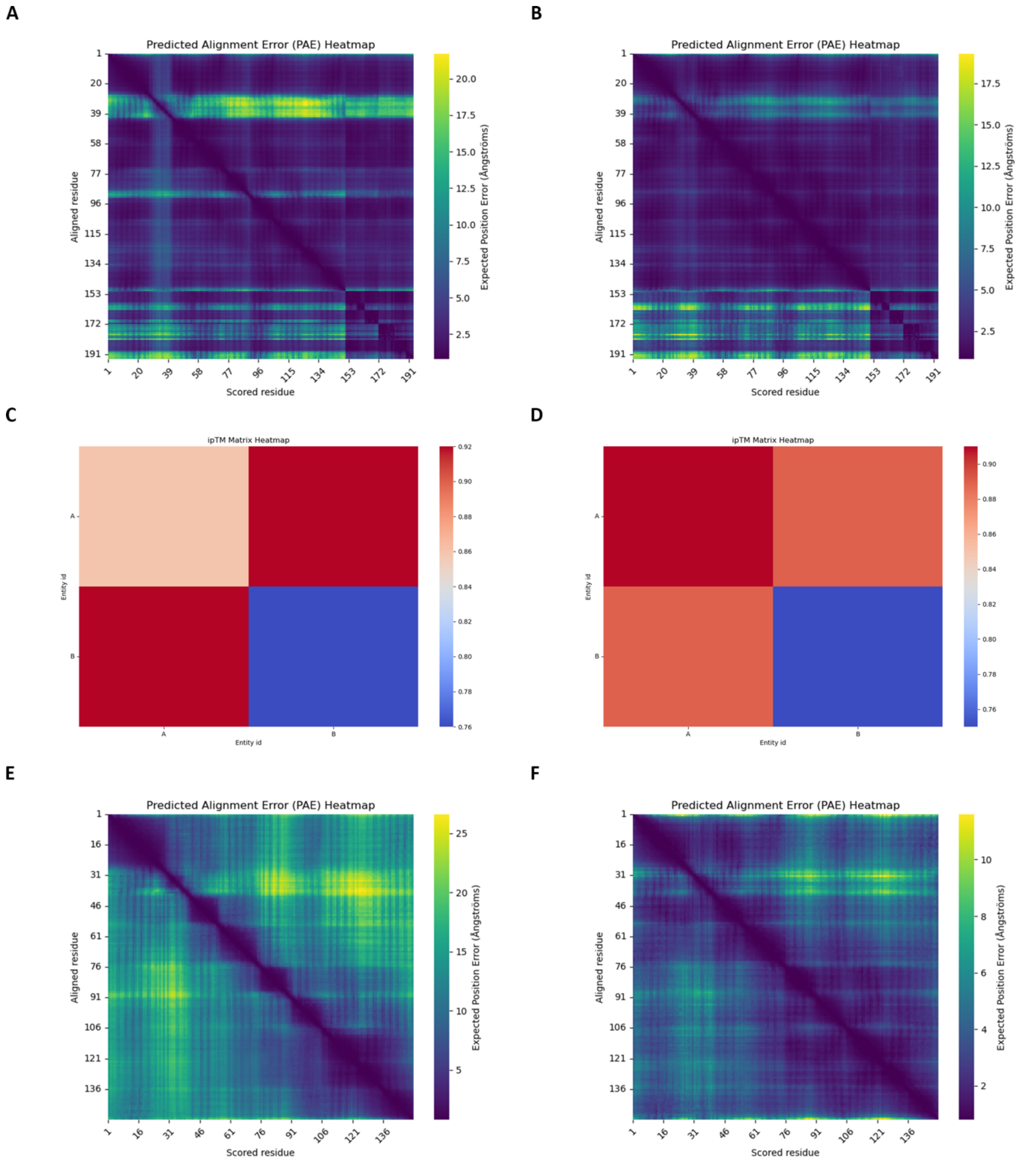

Fig. S12. PAE and iPTM plots for the apo- and holo- C11 and C11-620 AF3 models. (A, B) PAE plots for holo-C11 and holo-C11-620. Residues 150+ represent the PEB ligand atoms. (C, D) iPTM plots for holo-C11 and holo-C11-620 with PEB; chain A = protein, chain B = PEB. (E, F) PAE plots for apo-C11 and apo-C11-620.

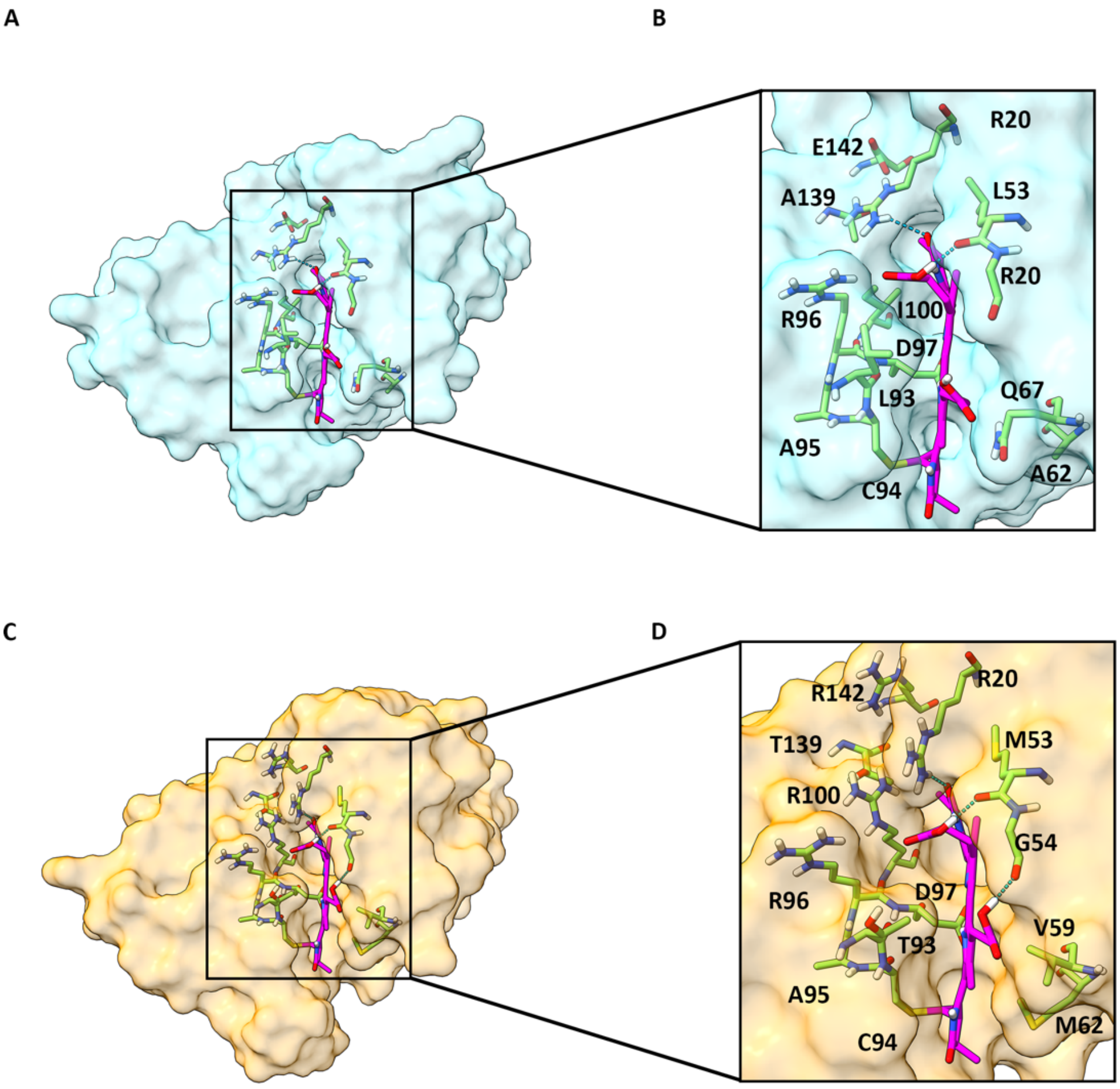

Fig. S13. Zoomed models of C11 (A-B) and C11-620 (C-D) highlighting the PEB-protein interactions and the CARD motif (residues 94-97). Hydrogen bonds are highlighted with cyan dotted lines.

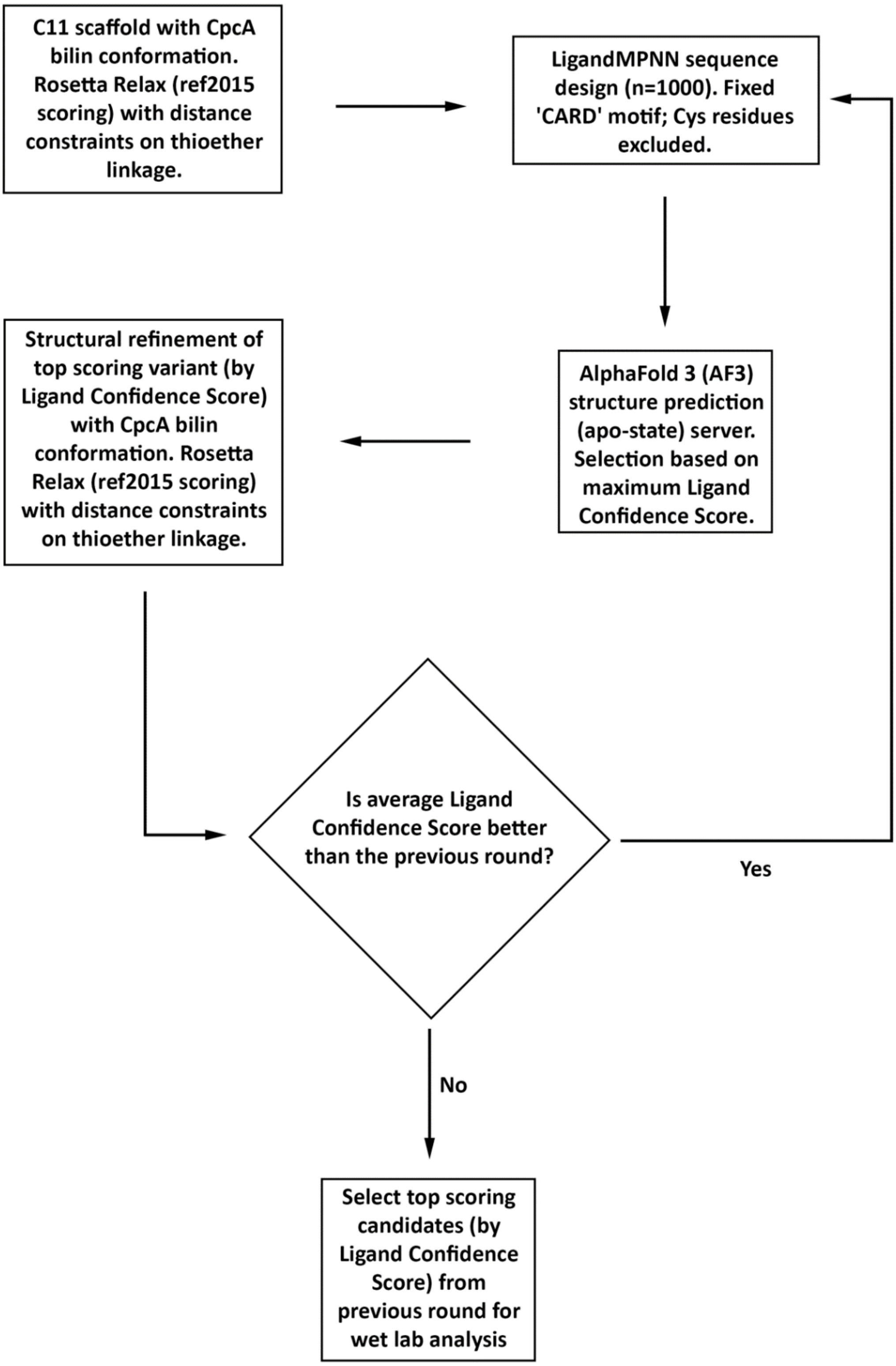

Fig. S14. Flowchart of the pipeline applied to the redesign of the C11 protein. The chart highlights the starting structure input generation, computational tools used and the decision point loop that results in the selection of the final design for wet lab testing.

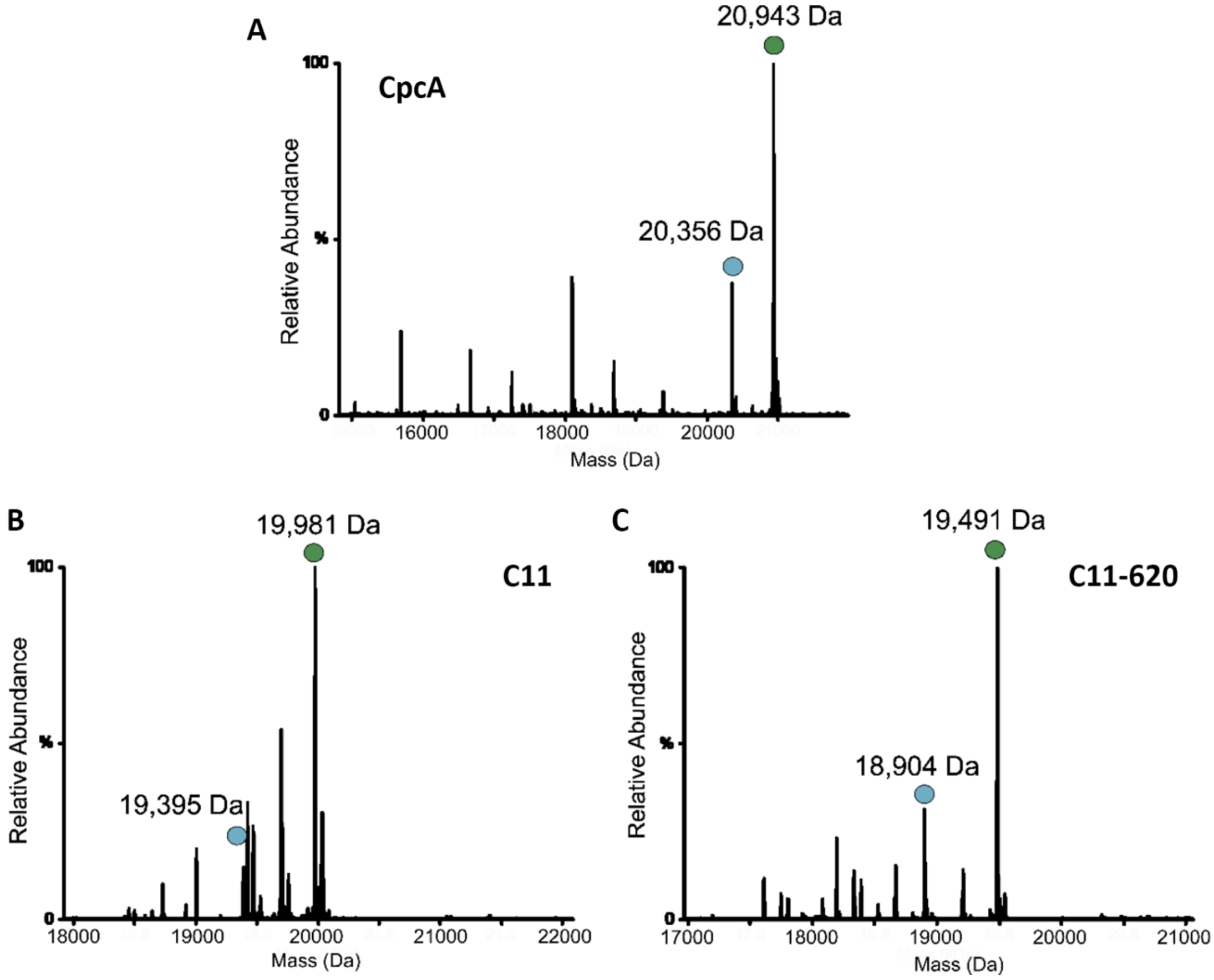

Fig. S15. Deconvoluted native mass spectra of (B) native CpcA, (B) the C11 design and (C) the C11-620 redesign. Peaks labelled in blue and green correspond to the unbound and bilin-bound complexes, respectively. Unlabelled peaks correspond to protein contaminants.

Table S1: Theoretical and observed molecular weights for most abundant unmodified and modified protein species as determined by native mass spectrometry.

Protein	Modification	Theoretical MW	Observed MW	Mass difference (Da)
CpcA	None	20,300.3 Da	20356.0 ± 0.7	+56 Da
CpcA	+PEB	20,887.0 Da	20943.2 ± 0.9	+56 Da
C11	None	19,334.7 Da	19,394.7 ± 2.1	+60 Da
C11	+PEB	19,921.4 Da	19,980.7 ± 0.9	+60 Da
C11-620	None	18,843.4 Da	18,903.8 ± 0.7	+60 Da
C11-620	+PEB	19,430.1 Da	19,490.5 ± 0.5	+60 Da

Table S2: Hydrogen bond donors to PEB in the original C11 and new designs

Design	Arg20 guanidinium to PEB D-ring keto oxygen	Met53 backbone carbonyl to PEB C-ring propanoic carboxyl	Gly54 backbone carbonyl to PEB B-ring propanoic carboxyl
C11	✓	✓	✗
C11-578	✗	✓	✓
C11-620	✓	✓	✓
C11-756	✗	✓	✓

Table S3: Description of the scoring components for the ref2015 scoring function

Scoring component name	Scoring component description
fa_atr	Lennard-Jones attractive between atoms in different residues
fa_rep	Lennard-Jones repulsive between atoms in different residues
fa_sol	Lazaridis-Karplus solvation energy
fa_intra_sol_xover4	Intra-residue Lazaridis-Karplus solvation energy
lk_ball_wtd	Asymmetric solvation energy
fa_intra_rep	Lennard-Jones repulsive between atoms in the same residue
fa_elec	Coulombic electrostatic potential with a distance-dependent dielectric
pro_close	Proline ring closure energy and energy of psi angle of preceding residue
hbond_sr_bb	Backbone-backbone hbonds close in primary sequence
hbond_lr_bb	Backbone-backbone hbonds distant in primary sequence
hbond_bb_sc	Sidechain-backbone hydrogen bond energy
hbond_sc	Sidechain-sidechain hydrogen bond energy
dslf_fa13	Disulfide geometry potential
rama_prepro	Ramachandran preferences (with separate lookup tables for pre-proline positions and other positions)
omega	Omega dihedral in the backbone. A Harmonic constraint on planarity with standard deviation of ~6 deg.
p_aa_pp	Probability of amino acid, given torsion values for phi and psi
fa_dun	Internal energy of sidechain rotamers as derived from Dunbrack's statistics
yhh_planarity	A special torsional potential to keep the tyrosine hydroxyl in the plane of the aromatic ring
ref	Reference energy for each amino acid. Balances internal energy of amino acid terms. Plays role in design.

Table S4: Rosetta Scores of the PEB ligand for C11 and C11-620. Major scoring components that increase stability are highlighted in yellow. Disulphide geometry potential (dslf_fa13) was omitted as the scores were zero for both C11 and C11-620.

	fa_atr	fa_rep	fa_sol	fa_intra_rep
C11-620	-25.0996	2.94266	0.53337	0.68338
C11	-23.7872	2.85299	1.8388	0.64122
Difference	-1.3124	0.08967	-1.30543	0.04216
	fa_intra_sol_xover4	lk_ball_wtd	fa_elec	pro_close
C11-620	1.69373	-0.30453	-3.99205	0
C11	1.51484	-0.94582	-2.94919	0
Difference	0.17889	0.64129	-1.04286	0
	hbond_sr_bb	hbond_lr_bb	hbond_bb_sc	hbond_sc
C11-620	0	0	-0.9411	-0.53399
C11	0	0	-0.51833	0
Difference	0	0	-0.42277	-0.53399
	atom_pair_constraint	omega	fa_dun	p_aa_pp
C11-620	-9.47227	0	0	0
C11	-9.47235	0	0	0
Difference	8.00E-05	0	0	0
	yhh_planarity	ref	rama_prepro	total_score
C11-620	0	0	0	-34.4904
C11	0	0	0	-30.8251
Difference	0	0	0	-3.6653

Table S5: Rosetta Scores of the C11 and C11-620 proteins, with their PEB ligands. Major scoring components that increase stability are highlighted in yellow. Disulphide geometry potential (dslf_fa13) was omitted as the scores were zero for both C11 and C11-620.

	fa_atr	fa_rep	fa_sol	fa_intra_rep
C11-620	-954.572	97.446	537.807	2.316
C11	-924.284	111.097	580.862	2.22
Difference	-30.288	-13.651	-43.055	0.096
	fa_intra_sol_xover4	lk_ball_wtd	fa_elec	pro_close
C11-620	29.926	-17.117	-320.261	0.577
C11	28.722	-23.74	-305.147	0.18
Difference	1.204	6.623	-15.114	0.397
	hbond_sr_bb	hbond_lr_bb	hbond_bb_sc	hbond_sc
C11-620	-99.346	-6.154	-21.716	-28.51
C11	-100.037	-8.023	-22.65	-22.099
Difference	0.691	1.869	0.934	-6.411
	atom_pair_constraint	omega	fa_dun	p_aa_pp
C11-620	-18.945	21.204	154.45	-33.405
C11	-18.945	9.937	175.036	-31.105
Difference	0	11.267	-20.586	-2.3
	yhh_planarity	ref	rama_prepro	total_score
C11-620	0.541	53.185	-7.085	-609.656
C11	0.255	14.582	-21.465	-554.603
Difference	0.286	38.603	14.38	-55.053
